# AI-Guided Ferroptosis Biomarker Discovery and Routine Laboratory–Based Machine Learning for Predicting Nodal Metastasis in Colon Cancer

**DOI:** 10.3390/metabo16080557

**Published:** 2026-08-06

**Authors:** Nail Besli, Talar Vartanoglu Aktokmakyan, Adil Koyuncu, Bahar Sarikamis Johnson, Ulkan Celik

**Affiliations:** 1 Department of Medical Biology, Hamidiye School of Medicine, University of Health Sciences, Istanbul 34668, Türkiye; beslinail@gmail.com; 2 General Surgery Department, Surp Pirgic Armenian Hospital, Istanbul 34020, Türkiye; talarim@gmail.com; 3 General Surgery Department, Istanbul Haseki Training and Research Hospital, University of Health Sciences, Istanbul 34130, Türkiye; dradilkoyuncu@gmail.com; 4 Department of Medical Biology, Faculty of Medicine, Istanbul Atlas University, Istanbul 34403, Türkiye; bahar.sarikamis@atlas.edu.tr

**Keywords:** colon cancer, ferroptosis, lymph node metastasis, machine learning, biomarkers, iron metabolism

## Abstract

**Background:** Accurate preoperative prediction of lymph node metastasis (LNM) remains challenging in colon cancer. Ferroptosis, an iron-dependent regulated cell-death pathway, is implicated in tumor invasion and metastasis. Yet, the clinical value of routine iron-metabolism indices as indirect systemic indicators of ferroptosis-relevant biology remains underexplored. **Methods:** A multi-stage workflow was implemented. First, transformer-based literature mining (PubMedBERT, BioLinkBERT, BioBERT) prioritized ferroptosis-associated genes and metabolites. Second, transcriptomic validation was performed in TCGA-COAD and GSE39582 cohorts using differential expression analysis. Third, machine-learning models (logistic regression, random forest, XGBoost) were developed to predict pathologically confirmed LNM (*n* = 421) under three feature configurations: preoperative baseline, preoperative plus iron-metabolism markers, and a pathology-augmented model. LVI prediction was separately evaluated (*n* = 416) using ferroptosis-only, clinical-only, and combined sets. **Results:** Literature mining identified a core ferroptosis axis dominated by redox and iron-handling regulators. Transcriptomic analyses demonstrated robust tumor–normal separation and consistent perturbation of key ferroptosis genes (SLC7A11, GPX4, ACSL4, PTGS2). Preoperative models achieved moderate LNM discrimination (best AUC = 0.704), whereas a postoperative pathology-augmented explanatory benchmark achieved an AUC of 0.818. Out-of-fold risk stratification identified a low-risk subgroup with 26.7% nodal positivity, compared with 44.7% in the overall cohort. For LVI prediction, models based on iron-handling indices showed modest discrimination, and their addition to clinical variables produced model-dependent numerical changes in performance (maximum ΔAUC = +0.114). These routinely measured indices provided exploratory predictive information but should not be interpreted as direct measures of tumor ferroptosis. **Conclusions:** This integrative framework links ferroptosis-related biology with clinical machine learning and suggests that routine iron-handling indices may provide exploratory complementary information for LVI prediction and preoperative nodal-risk stratification, whereas postoperative pathology variables provide additional explanatory value but are not part of a deployable preoperative model.

## 1. Introduction

Colon cancer remains one of the most prevalent malignancies worldwide, ranking as the third-most diagnosed cancer and the second leading cause of cancer-related mortality, with an estimated 1.9 million new cases and 935,000 deaths reported globally in 2020 [1]. Despite significant advances in surgical techniques and adjuvant therapies, lymph node metastasis remains a critical prognostic factor that profoundly influences treatment decisions and patient outcomes [2]. Accurate preoperative prediction of lymph node involvement is essential for risk stratification, surgical planning, and determining the need for neoadjuvant therapy [3].

Traditional staging methods, including computed tomography (CT) and magnetic resonance imaging (MRI), demonstrate limited sensitivity (60–75%) in detecting lymph node metastasis, particularly for micro metastases that significantly impact prognosis [4,5]. This diagnostic gap has prompted the search for novel biomarkers and predictive models that can complement imaging-based staging. Recent advances in systems biology and molecular oncology have revealed that cancer progression is driven by complex interactions between multiple biological pathways, including metabolic reprogramming, immune evasion, and regulated cell death mechanisms [6].

Ferroptosis, an iron-dependent form of regulated cell death characterized by lipid peroxidation and glutathione depletion, has emerged as a critical mechanism in cancer biology [7]. Unlike apoptosis or necrosis, ferroptosis is driven by dysregulation of iron metabolism and oxidative stress, with key regulatory proteins including glutathione peroxidase 4 (GPX4), solute carrier family 7 member 11 (SLC7A11), and acyl-CoA synthetase long-chain family member 4 (ACSL4) [8,9]. Accumulating evidence suggests that ferroptosis dysregulation is associated with tumor progression, metastatic phenotypes, and therapeutic resistance in various malignancies, including colon cancer [10,11].

The relationship between ferroptosis and cancer metastasis is particularly intriguing. Iron metabolism dysregulation has been implicated in epithelial–mesenchymal transition (EMT), a critical process in cancer invasion and metastasis [12]. Because ferroptosis regulation intersects with iron handling, redox balance, and EMT-associated programs, ferroptosis dysregulation may plausibly be associated with invasive phenotypes such as lymphovascular invasion (LVI) and with an increased risk of nodal spread [13,14]. However, the clinical utility of ferroptosis biomarkers in predicting lymph node metastasis remains underexplored, and routine laboratory markers of iron metabolism—such as hemoglobin, mean corpuscular volume (MCV), serum iron, total iron-binding capacity (TIBC), and ferritin—have not been systematically evaluated in this context. Additionally, routine iron indices reflect systemic iron availability, anemia patterns, and inflammation-associated iron sequestration—processes that overlap with ferroptosis biology and tumor–host metabolic crosstalk—making them attractive, low-cost candidates for preoperative risk modeling.

The integration of artificial intelligence (AI) and machine learning (ML) into oncology has revolutionized biomarker discovery and clinical prediction [15]. Deep learning models, particularly transformer-based architectures trained in biomedical literature, have demonstrated remarkable capabilities in extracting complex relationships from vast amounts of unstructured data [16,17]. These models can identify novel biomarker candidates by analyzing millions of publications, clinical trials, and molecular databases, accelerating the translation of basic science discoveries into clinical applications [18].

In parallel, machine learning algorithms have shown exceptional performance in predicting clinical outcomes using routine laboratory data, offering practical tools for risk stratification without requiring expensive molecular profiling [19]. Random forest, gradient boosting, and deep neural networks have been successfully applied to predict lymph node metastasis, treatment response, and survival in various cancers [20,21]. However, most studies have focused on traditional clinical parameters or imaging features, with limited exploration of ferroptosis-related biomarkers.

Despite these advances, several critical gaps remain in our understanding of ferroptosis in colon cancer metastasis. First, few studies have jointly integrated AI-guided biomarker discovery with transcriptomic validation and clinical machine learning to establish a comprehensive ferroptosis signature for lymph node metastasis prediction. Second, the biological relationship between ferroptosis dysregulation, lymphovascular invasion, and subsequent lymph node metastasis has not been clearly delineated. Third, the clinical utility of routine laboratory markers of iron metabolism—which are readily available in standard preoperative workups—has not been fully exploited for risk stratification.

In this study, we developed an integrated ferroptosis-oriented framework for preoperative risk stratification in colon cancer. First, transformer-based literature mining was used to prioritize ferroptosis-associated biomarker candidates linked to invasion and metastasis. Second, candidate signals were validated using independent transcriptomic cohorts. Third, machine-learning models were trained on routinely available preoperative laboratory indices—including iron metabolism parameters—to predict lymphovascular invasion and to evaluate preoperative nodal-risk stratification; a pathology-augmented model was additionally constructed to quantify the explanatory contribution of postoperative histopathological variables. This integrative strategy aims to bridge ferroptosis-related biological evidence and practical clinical decision support, clearly delineating the utilities of preoperative and postoperative models.

## 2. Materials and Methods

### 2.1. Study Design and Overall Workflow

**Figure 1:**
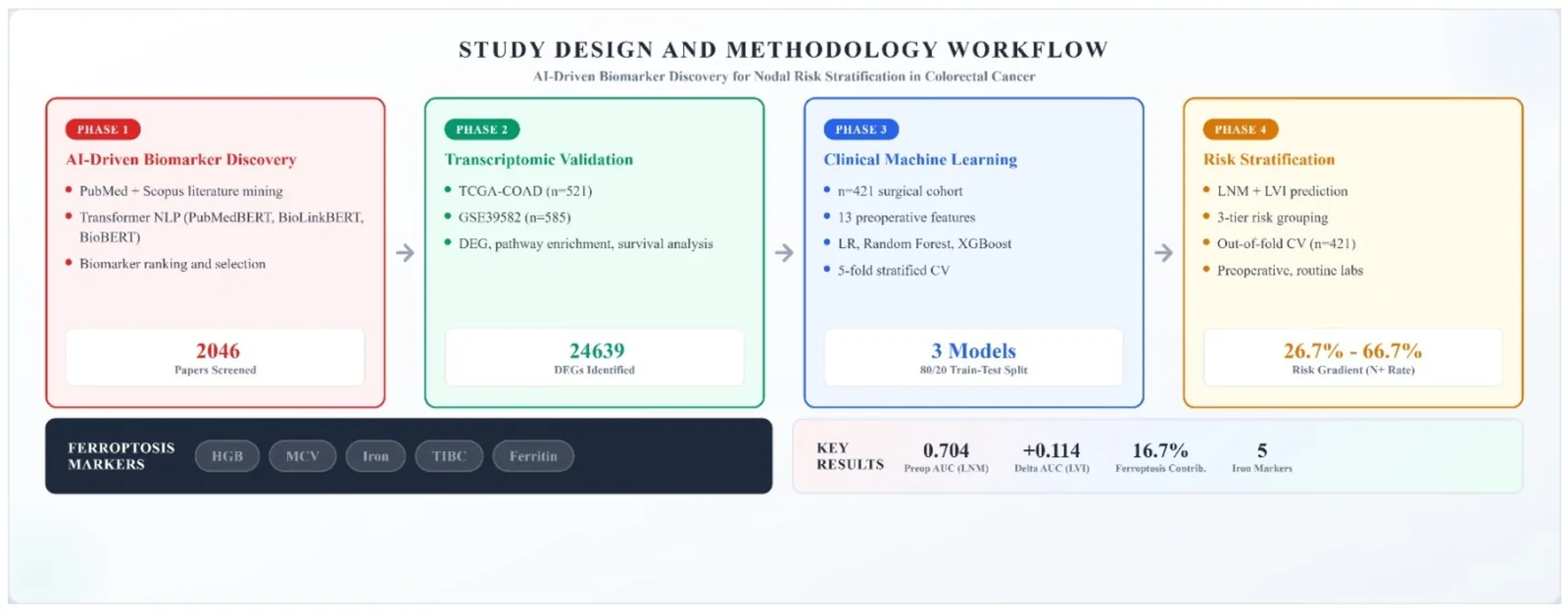
Study design and methodology workflow.

This study employed a multi-stage integrative framework to identify ferroptosis-associated biomarkers in colon cancer, validate findings across independent cohorts, and translate them into a clinical prediction model (Figure 1). The workflow comprised four sequential phases: (i) deep learning-assisted literature mining of 2046 publications retrieved from PubMed/MEDLINE and Scopus using transformer-based NLP models (PubMedBERT, BioLinkBERT, BioBERT) to rank candidate biomarkers; (ii) differential gene expression analysis and ferroptosis signature interrogation in the TCGA-COAD RNA-seq cohort (*n* = 500); (iii) independent transcriptomic validation in the GEO microarray cohort GSE39582 (*n* = 585), including pathway enrichment and survival analyses; and (iv) clinical machine-learning model development and preoperative risk stratification in a surgical cohort of 421 patients using logistic regression, random forest, and XGBoost classifiers with 5-fold stratified cross-validation. Analyses were conducted using open-source Python (v 3.11) and R (v 4.4.2) environments. Google Colab was used for substantial portions of the literature-mining workflows; clinical machine learning analyses were implemented locally using scikit-learn (v 1.3) and XGBoost (v 2.0).

The disease scope of the present study was colon cancer. The institutional cohort was restricted to histologically confirmed colon adenocarcinoma, TCGA analyses were restricted to TCGA-COAD, and no TCGA-READ samples were included. The term “colon cancer” is used only when referring to external studies that explicitly included both colon and rectal cancers.

### 2.2. AI-Guided Literature Mining

#### 2.2.1. Data Sources and Search Strategy

To identify candidate ferroptosis-related biomarkers linked to colon cancer, we systematically searched PubMed/MEDLINE and Scopus. Searches were automated using Python scripts executed in Google Colab. Colon cancer-related terms included “colon cancer,” “colon cancer,” “CRC,” “colon carcinoma,” “colon adenocarcinoma,” and “colon neoplasm.” Ferroptosis-related terms included “ferroptosis,” “iron-dependent cell death,” “lipid peroxidation,” “GPX4,” “SLC7A11,” “FSP1,” “oxidative stress,” and “reactive oxygen species.” Queries were assembled using a logical conjunction of concepts, i.e., (colon cancer terms) AND (ferroptosis terms), to capture publications at the intersection of both domains.

For each retrieved record, bibliometric metadata were programmatically extracted, including title, abstract (when available), authorship, publication year, journal name, DOI, PubMed ID, Scopus ID, author keywords, and citation counts. Data acquisition and structuring were performed using standard Python libraries (e.g., requests, pandas) and NCBI/Entrez-compatible utilities where applicable, producing structured tables for downstream analysis.

#### 2.2.2. Transformer Models and Embedding Extraction

Three biomedical transformer models were used to represent and score biomarker evidence from text:

PubMedBERT (microsoft/BiomedNLP-PubMedBERT-base-uncased-abstract-fulltext), trained on PubMed abstracts and full texts, generating 768-dimensional embeddings.

BioLinkBERT (michiyasunaga/BioLinkBERT-large), trained using citation/link structures in biomedical literature, generating 1024-dimensional embeddings.

BioBERT (dmis-lab/biobert-v1.1), trained on PubMed/PMC corpora and optimized for biomedical semantic representation, generating 768-dimensional embeddings [16].

Although these models overlap in their biomedical scope, they differ in their pretraining strategies and therefore provide partly complementary representations of biomedical evidence. PubMedBERT was trained from scratch exclusively on biomedical literature and served as the principal domain-specific representation model. BioBERT represents a general-domain BERT model further pretrained on PubMed and PMC corpora, whereas BioLinkBERT additionally incorporates citation-link information and may capture relationships distributed across interconnected publications. The multi-model design was therefore used as a robustness and consensus strategy to reduce dependence on the embedding geometry and pretraining characteristics of any single transformer, rather than assuming that using three models necessarily outperforms PubMedBERT alone.

Abstract texts were cleaned to remove non-informative special characters and were tokenized using each model’s tokenizer with a maximum sequence length of 512 tokens. For each record, a single document-level embedding was computed by pooling the final hidden states (mean pooling) to obtain a fixed-length vector representation. Cosine-similarity scores were calculated separately within each model-specific embedding space because the raw embeddings generated by the three models differed in dimensionality and were not directly comparable. The resulting model-derived semantic evidence was incorporated into the evidence-strength component of the composite prioritization framework described in Section 2.2.4. Thus, the transformer models constituted one evidence layer within the overall prioritization process rather than independent predictive endpoints. Computations were accelerated on a GPU when available (e.g., A100 on Google Colab); otherwise, processing was conducted on a CPU.

#### 2.2.3. Gene/Protein Name Extraction and Candidate List Generation

Candidate gene and protein mentions were extracted from text using a hybrid strategy combining regular expression-based pattern matching and biomedical term normalization heuristics. For gene symbols, an HGNC-like uppercase pattern (e.g., A–ZA–Z0–9{1,10}) was used as a primary filter, followed by context-based screening to reduce false positives. Candidate entities were then summarized by frequency statistics and TF–IDF metrics. In parallel, cosine similarity between document embeddings and candidate entity representations was computed to quantify contextual alignment with colon cancer–ferroptosis concepts. Enrichment of candidate mentions in ferroptosis-focused contexts was assessed using statistical tests (e.g., Fisher’s exact test), with multiple-testing correction performed using the Benjamini–Hochberg false discovery rate (FDR) procedure.

#### 2.2.4. Definition of a Core Ferroptosis Gene Panel

Candidate genes were assigned a composite prioritization score (CS) calculated as a weighted linear combination of five normalized sub-scores: evidence strength (30%), statistical significance (25%), clinical evidence density (20%), effect magnitude (15%), and cross-study reliability (10%). Each component was min–max normalized to a 0–100 scale before weighting. The evidence component integrated study-type weighting, transformer-derived semantic similarity, and ferroptosis-specific textual relevance; the statistical component reflected FDR-corrected enrichment; the clinical component captured the density of clinical-study evidence; the effect component represented standardized signal magnitude; and the reliability component reflected consistency across retrieved studies. The candidate universe was predefined using established ferroptosis regulators, ferroptosis-associated pathway genes, colon cancer drivers, and curated entity synonyms. Thus, canonical candidates were seeded rather than discovered de novo. The transformer-assisted workflow retrieved, contextualized, and ranked corpus-derived evidence within this predefined candidate space; it did not perform unrestricted gene discovery. Accordingly, the DL-Top 15 represents a literature-prioritized subset of predefined candidates rather than an independently AI-discovered gene signature.

Transformer-derived semantic relevance was evaluated separately within the embedding space of each model. Evidence supported across these alternative biomedical representations was used to reduce dependence on a single model’s pretraining characteristics. Accordingly, the multi-model component functioned as a robustness-oriented evidence layer within the composite score. Because a formal comparison between the multi-model strategy and PubMedBERT alone was not performed, this approach was not interpreted as demonstrating superior performance over a single-model strategy.

The weighting scheme was designed to prioritize biologically interpretable and clinically supported ferroptosis candidates, while avoiding reliance on a single metric. The DL-Top 15 panel was therefore used as a high-confidence prioritization set for downstream transcriptomic validation rather than as a definitive list of causal biomarkers.

Based on these composite scores, candidate genes were stratified into three categories: (i) Core ferroptosis genes (DL-Top 15)—a high-confidence panel defined by centrality in ferroptosis biology and high text-derived evidence scores (GPX4, SLC7A11, ACSL4, FSP1/AIFM2, NFE2L2/NRF2, PTGS2/COX2, ALOX12, ALOX5, TP53, KRAS, BRAF, FTH1, ATF4, TFRC, and CAT). (ii) Ferroptosis regulatory genes that indirectly modulate ferroptotic processes (e.g., KEAP1, HMOX1, NCOA4, LPCAT3, ALOX15, ATF3, CHAC1, HSPB1, CISD1/2, GLS2, SAT1, VDAC2/3, DPP4). (iii) Ferroptosis-associated pathway components, including autophagy-related genes (e.g., BECN1, ATG5, ATG7), transcriptional regulators (e.g., STAT3, YAP1, BAP1), and oncogenic pathway genes (e.g., APC, PIK3CA, SMAD4). Ranked gene lists and the DL-Top 15 core panel were exported (e.g., ferroptosis_genes_ranked) for use in TCGA and GEO analyses. Complete database-specific search strings, retrieval dates, study-type classification rules, candidate dictionaries, entity-harmonization procedures, scoring equations, and executable scripts are provided in the Supplementary Methods and Supplementary Code.

### 2.3. TCGA-COAD Transcriptomic Analysis

#### 2.3.1. Data Source and Preprocessing

RNA-seq expression profiles for colon adenocarcinoma were obtained from the TCGA-COAD project via cBioPortal (PanCancer Atlas). The downloaded expression matrix was provided in RSEM-normalized log2(RSEM + 1) format and covered approximately 20,000 protein-coding genes. The cohort comprised 459 tumor samples and 41 normal colon mucosa samples (total *n* = 500). Clinical variables, including age, sex, stage, MSI status, and available survival fields, as well as somatic mutation data in MAF format, were also retrieved.

Data ingestion and preprocessing were performed in R (v. 4.4.2) using standard utilities, including data.table and readr. Tumor/normal status was assigned from TCGA barcode sample-type codes, with codes 01–09 classified as tumor and codes 10–19 classified as normal. Because the cBioPortal-derived matrix was available in log2-transformed RSEM format rather than as raw HTSeq/GDC integer counts, an explicit back-transformation step was applied before count-based differential expression modeling. Specifically, expression values were transformed using 2^expr − 1 and rounded to generate approximate count-like values. Negative values, when present as normalization artifacts, were set to zero before transformation. Low-expression genes were filtered using a minimal abundance criterion of total count ≥10 across samples.

#### 2.3.2. Differential Expression Analysis

Differential expression between tumor and normal samples was performed using DESeq2 with the Wald test. Genes were considered significant if FDR < 0.05 and |log2 fold-change| > 0.5, balancing statistical confidence with biological effect size. Result tables included log2 fold-change, standard errors, Wald statistics, raw *p*-values, and Benjamini–Hochberg-adjusted *p*-values.

We acknowledge that DESeq2 is ideally applied to un-normalized integer count matrices and that raw HTSeq/GDC counts would represent the preferred input format. Therefore, the TCGA-COAD analysis based on back-transformed RSEM values was interpreted as an approximate transcriptomic validation layer rather than as a stand-alone definitive raw-count RNA-seq analysis. To reduce dependence on this preprocessing choice, ferroptosis-related expression patterns were evaluated together with the independent GSE39582 cohort, which was analyzed separately using the limma framework appropriate for microarray data.

#### 2.3.3. Visualization and Ferroptosis Gene Integration

TCGA differential expression results were visualized using ggplot2, ggrepel, and pheatmap. Volcano plots displayed log2 fold-change versus −log10(FDR), with thresholds (|log2FC| = 0.5; FDR = 0.05) indicated by dashed lines. Significant upregulated genes were colored in red, down-regulated genes in blue, and non-significant genes in gray. Ferroptosis-associated genes were overlaid on volcano plots; DL-Top 15 genes were highlighted using distinct marker/label styling relative to other ferroptosis-related genes.

Additionally, the top 50 genes ranked by absolute log2 fold-change were selected for heatmap visualization. Expression values were Z-score normalized across genes (row-wise), and samples were annotated by Tumor/Normal status. PCA was performed on Z-scored expression matrices derived from significant DEGs using the R prcomp function, and separation between groups was evaluated in the PC1–PC2 space. This DEG-based PCA was used as an exploratory visualization rather than an independent validation analysis.

### 2.4. Independent GEO Validation: GSE39582

#### 2.4.1. Dataset and Phenotype Annotation

Independent validation was carried out using the GEO dataset GSE39582, generated on the GPL570 platform (Affymetrix Human Genome U133 Plus 2.0). The dataset contains 585 samples, including 566 colon cancer tissues and 19 normal colon mucosa samples. Associated clinical annotations include stage, grade, MSI status, and survival variables where available.

Data were downloaded in R (v. 4.4.2) using GEOquery, and expression/phenotype tables were processed separately. Tumor versus normal classification was derived from phenotype fields (e.g., source_name_ch1), mapping “normal mucosa/non tumoral mucosa” to the normal group and “adenocarcinoma/carcinoma/cancer” to the tumor group.

#### 2.4.2. Differential Expression Analysis (Limma)

Microarray differential expression was performed using limma. After RMA normalization (and quantile normalization when required), a design matrix contrasting tumor and normal groups was constructed. Linear modeling and empirical Bayes moderation were applied to compute logFC, *p*-values, and adjusted *p*-values (Benjamini–Hochberg). Probe identifiers were mapped to HGNC gene symbols using platform annotation. Non-informative records (e.g., lacking gene symbols or representing ambiguous/non-coding entities such as LINC/LOC/MIR) were removed. For genes represented by multiple probes, a single representative probe was selected based on statistical strength (lowest adjusted *p*-value and highest absolute logFC), yielding a gene-level DEG table focused on protein-coding genes. Significance criteria were set to adjusted *p*-value < 0.05 and |logFC| > 0.5.

#### 2.4.3. Visualization in GSE39582

The visualization strategy used for TCGA was applied consistently to GSE39582. Volcano plots were generated with ferroptosis genes annotated and DL-Top 15 genes highlighted. Heatmaps were created using the top 50 genes ranked by absolute logFC with row-wise Z-score normalization and Tumor/Normal sample annotation. PCA was conducted on DEG-based expression matrices to assess tumor–normal separation in principal component space, providing an additional quality-control and interpretability layer. Collectively, this approach enabled platform- and cohort-independent evaluation of TCGA-derived findings.

### 2.5. Statistical Analysis

For TCGA-COAD, differential expression was tested using DESeq2 (Wald test), and for GSE39582, limma moderated *t*-tests were applied. In both cohorts, multiple-testing correction used the Benjamini–Hochberg method, with FDR/adjusted *p*-value < 0.05 considered statistically significant. To improve biological interpretability, an effect-size threshold of |log2FC| > 0.5 was also applied.

Heatmaps used row-wise Z-score normalization, Euclidean distance, and complete-linkage clustering. PCA was performed on Z-scored expression matrices using the R prcomp function, and explained variance ratios were reported.

### 2.6. Software and Computational Environment

AI-guided literature mining and embedding computation were implemented in Python 3, primarily on Google Colab. Key Python libraries included transformers, torch, pandas, scipy, statsmodels, matplotlib, and seaborn. Transcriptomic analyses were conducted in R (v. 4.4.2), using DESeq2, limma, GEOquery, ggplot2, ggrepel, pheatmap, RColorBrewer, and dplyr. To ensure reproducibility, fixed random seeds (seed = 42) were used in both Python and R environments where applicable. Clinical machine-learning analyses were implemented in Python 3.10 using scikit-learn (v1.3), XGBoost (v2.0), and imbalanced-learn; all pipelines were constructed with scikit-learn Pipeline objects.

### 2.7. Data Management and Quality Control

Raw and processed datasets were stored systematically in both Google Drive and local project directories. Public transcriptomic datasets (TCGA, GEO) were subjected to standard platform-specific QC, supplemented by PCA and hierarchical clustering to evaluate potential outliers and batch artifacts visually. For microarray data, non-coding/control probes were removed, gene symbols were standardized, and representative probes were selected to improve gene-level interpretability.

Because TCGA and GEO datasets are publicly available and de-identified, no additional patient consent or local ethical approval was required for their use. Bibliometric metadata retrieved from PubMed and Scopus were processed in compliance with the respective API usage policies, and no personally identifiable information was collected or analyzed.

### 2.8. Clinical Cohort and Endpoint Definitions

A retrospective single-center cohort of patients who underwent curative-intent surgical resection for histologically confirmed colon adenocarcinoma at Koşuyolu Yüksek İhtisas Training and Research Hospital was assembled. From an initial registry of 704 patients, those with missing or indeterminate pathological lymph node status were excluded, yielding a final analytic cohort of 421 patients for LNM modeling (male: 249; female: 172). A parallel subcohort of 416 patients with available LVI annotations was used for LVI prediction (male: 246; female: 170).

The primary endpoint for LNM modeling was pathologically confirmed lymph node metastasis (N_Status), defined as the presence of at least one metastatic lymph node in the surgical specimen on final histopathological examination. The primary endpoint for LVI modeling was pathologist-reported lymphovascular invasion. Both endpoints are postoperative, histopathology-derived variables; the clinical objective was therefore to evaluate whether preoperatively available laboratory indices can predict these pathologically determined outcomes.

### 2.9. Predictor Variables and Feature Configurations

Three progressively enriched feature configurations were defined for LNM prediction:

(i) Preoperative baseline (8 features): age, CEA, CA 19-9, neutrophil count, lymphocyte count, platelet count, albumin, and LDH—all obtainable from standard preoperative workup.
(ii) Preoperative baseline + ferroptosis/iron-metabolism markers (13 features): the baseline panel augmented with hemoglobin, MCV, serum iron, total iron-binding capacity (TIBC), and ferritin. These five indices were selected as clinical proxies for ferroptosis-related iron handling based on the AI-driven literature-mining results (Section 2.2) and are routinely measured in preoperative laboratory panels.
(iii) Pathology-augmented model (15 features): the 13-feature preoperative panel supplemented with LVI and total harvested lymph node count—both derived from postoperative histopathological examination. This configuration was included as an explanatory benchmark to quantify the additional discriminative contribution of pathology variables; it is not intended for preoperative clinical deployment.

For LVI prediction, three separate feature configurations were evaluated: ferroptosis markers only (5 features), clinical variables only (8 features), and a combined clinical + ferroptosis set (13 features). All predictor variables in the LVI task are preoperatively available.

### 2.10. Data Preprocessing

Preprocessing was embedded within scikit-learn Pipeline objects to prevent information leakage between training and test partitions. Missing values were imputed using median imputation (SimpleImputer, strategy = ‘median’), fitted exclusively on the training set and applied to the test set via transform. Missingness was substantial for selected tumor-marker and iron-handling variables; complete variable-level rates, outcome-stratified comparisons, and missingness-exclusion sensitivity analyses are described in Missing Data Handling and Sensitivity Analyses and Supplementary Tables S2 and S3. Continuous features were standardized using z-score normalization (StandardScaler) for logistic regression; tree-based models (random forest, XGBoost) were trained without scaling. No log-transformation or outlier removal was applied. Categorical variables were already binary-coded in the source dataset and required no additional encoding.

#### Missing Data Handling and Sensitivity Analyses

Because this was a retrospective cohort spanning approximately ten years, selected laboratory variables were not uniformly measured under a standardized prospective protocol. Iron-metabolism markers and tumor markers were requested selectively during routine clinical care, whereas routine hematology parameters were more consistently available. Within the final LNM cohort (*n* = 421), missingness was 74.8% for ferritin, 72.2% for serum iron, 70.5% for TIBC, 54.2% for CA19-9, 47.3% for CEA, and 27.8% for hemoglobin. Only 40 patients (9.5%) had complete observations across all 13 predictors included in the preoperative iron-handling model.

Little’s MCAR test rejected the hypothesis that the data were missing completely at random (χ^2^ = 550.94, df = 421, *p* < 0.0001), consistent with selective laboratory ordering in retrospective clinical data. To assess whether missingness was directly associated with the study endpoint, variable-level missingness was tested against lymph node metastasis status. No significant associations were observed for the evaluated variables, including ferritin (OR = 1.02, *p* = 1.000), CEA (OR = 1.22, *p* = 0.363), and CA19-9 (OR = 0.93, *p* = 0.796), supporting missing-at-random as a working assumption. Missingness was additionally evaluated separately in N− and N+ patients using variable-level 2 × 2 tables and Fisher’s exact tests. Missingness rates were comparable between the outcome groups for ferritin (74.7% vs. 75.0%), serum iron (71.7% vs. 72.9%), TIBC (71.2% vs. 69.7%), CA19-9 (54.9% vs. 53.2%), and CEA (45.1% vs. 50.0%); none of these comparisons was statistically significant (all *p* > 0.05).

For the primary analysis, missing values were handled using median imputation within scikit-learn Pipeline objects to avoid information leakage. Sensitivity analyses compared the primary median-imputation approach with K-nearest-neighbor imputation, MICE-style iterative imputation using BayesianRidge, and MICE-style iterative imputation using random forest. These imputation-sensitivity analyses were conducted using the same 13-predictor preoperative panel comprising age, CEA, CA19-9, neutrophil count, lymphocyte count, platelet count, albumin, LDH, hemoglobin, MCV, serum iron, TIBC, and ferritin. Median imputation yielded equal or higher discrimination compared with alternative methods. In the random forest model, test AUCs were 0.663 for median imputation, 0.602 for KNN, 0.629 for MICE-BayesianRidge, and 0.662 for MICE-RF. In the XGBoost model, test AUCs were 0.708, 0.616, 0.632, and 0.634, respectively. Feature-importance rankings were perfectly concordant across imputation strategies (Spearman ρ = 1.000), indicating that predictor-contribution patterns were robust to the imputation approach. Complete-case analysis was not considered sufficiently reliable because only 40 of the 421 patients (9.5%) had complete observations across all predictors in the final 13-predictor LNM panel. Because complete-case analysis would cause substantial information loss and unstable estimation, it was not used as confirmatory evidence. Analyses involving variables with excessive missingness were considered exploratory.

To determine whether model performance was driven by variables with excessive missingness, an additional exclusion sensitivity analysis was performed using a prespecified threshold of ≥50% missingness. CA19-9, serum iron, TIBC, and ferritin were excluded, yielding a reduced nine-predictor panel comprising age, CEA, neutrophil count, lymphocyte count, platelet count, albumin, LDH, hemoglobin, and MCV. A more restrictive six-predictor panel containing only variables with ≤30% missingness—age, neutrophil count, lymphocyte count, platelet count, hemoglobin, and MCV—was also evaluated. All sensitivity models retained the same LNM cohort, stratified 80:20 train–test partition, random seed, training-only median-imputation procedure, and model hyperparameters used in the primary analysis. Outcome-stratified missingness is reported in Supplementary Table S2, whereas the predictor definitions and performance results for the exclusion sensitivity analyses are provided in Supplementary Table S3.

### 2.11. Model Development and Validation

Data were partitioned into 80% training and 20% test sets using stratified random splitting (random_state = 42) to preserve class balance. Three classifiers were evaluated:

Logistic regression (LR): penalty = L2, C = 1.0, solver = lbfgs, max_iter = 1000.

Random forest (RF): n_estimators = 200, max_depth = 6, min_samples_split = 20 (LNM task); n_estimators = 100, default depth (LVI task).

XGBoost: n_estimators = 100, max_depth = 4, learning_rate = 0.1, eval_metric = logloss (LNM task); n_estimators = 100, default settings (LVI task).

Internal validation employed repeated stratified K-fold cross-validation (5 folds × 10 repeats = 50 iterations) on the training set, with AUC-ROC as the scoring metric. Final model performance was assessed on the held-out test set. Additional methodological sensitivity analyses were performed to evaluate class imbalance handling and hyperparameter selection. The LNM training set showed only moderate class imbalance, with an N−/N+ ratio of 1.24. Repeated stratified K-fold cross-validation was therefore used to preserve class proportions across folds. Class-weighted alternatives were additionally evaluated. Class weighting did not improve random forest performance (test AUC: 0.663 without weighting vs. 0.660 with balanced class weights) and produced only a modest improvement for XGBoost (test AUC: 0.708 without weighting vs. 0.729 with scale_pos_weight). Given the moderate imbalance and limited sample size, synthetic oversampling was not applied to avoid introducing artificial observations into the clinical dataset.

To assess whether model performance was limited by manual hyperparameter selection, GridSearchCV-based sensitivity analysis was performed. For random forest, GridSearchCV produced the same test AUC as the manually selected configuration (0.663 vs. 0.663). For XGBoost, the manually selected configuration outperformed the GridSearchCV-selected model (0.708 vs. 0.655). These findings suggest that the manually selected hyperparameters were reasonable for the cohort size and feature set and did not materially limit model performance.

Moreover, uncertainty in held-out performance was quantified using 1000 stratified bootstrap resamples to derive percentile-based 95% confidence intervals. Nested cross-validation was additionally performed as a sensitivity analysis to assess the stability of AUC estimates. Detailed bootstrap confidence intervals for all held-out performance metrics are provided in Supplementary Table S4 and Figure S9.

### 2.12. Performance Metrics and Model Interpretability

Discrimination was assessed using the area under the receiver operating characteristic curve (AUC-ROC) as the primary metric; accuracy, precision, recall, and F1-score were reported as secondary metrics for LNM models. Uncertainty in held-out performance was quantified using 1000 nonparametric bootstrap resamples of the paired outcome–prediction observations to derive percentile-based 95% confidence intervals.

The incremental contribution of iron-handling indices to LNM discrimination was formally evaluated by comparing paired held-out predictions from the eight-predictor baseline and 13-predictor augmented models. AUC differences were assessed using two-sided paired DeLong tests, and 95% confidence intervals for ΔAUC were estimated using 10,000 paired-bootstrap resamples.

Feature importance was evaluated using three complementary approaches: impurity-based mean decrease in Gini, model-agnostic permutation importance, and SHAP values. Permutation importance was calculated on the held-out test set using 100 repetitions and AUC as the scoring metric. SHAP values were calculated using TreeExplainer to evaluate the magnitude and direction of individual predictor contributions.

Calibration was evaluated using Brier scores and calibration curves. Brier scores were 0.228 for random forest and 0.217 for XGBoost; these values indicate only limited improvement over the null-model Brier score of 0.247 and should not be interpreted as evidence of strong calibration. Decision-curve analysis was used as an exploratory assessment of net benefit across threshold probabilities; these internally derived findings were not interpreted as evidence of established clinical utility.

Multicollinearity was assessed using variance inflation factors. Ferritin showed low multicollinearity (VIF = 1.49), whereas MCV (VIF = 28.96) and age (VIF = 19.71) showed higher values. However, the primary random forest and XGBoost models are less sensitive to multicollinearity than coefficient-based linear models because they rely on recursive partitioning and ensemble learning. Detailed results on missingness, validation, incremental value, and SHAP are provided in Supplementary Tables S2–S4 and Figures S7–S9.

### 2.13. Risk Stratification

For the LNM prediction task, out-of-fold predicted probabilities were generated via 5-fold stratified cross-validation using the preoperative model (baseline + ferroptosis features, random forest) so that every patient received a risk score from a model that excluded them during training. Patients were assigned into three risk tiers: low (predicted probability < 0.30), medium (0.30–0.70), and high (>0.70). These thresholds were selected a priori and were not optimized on outcome data. Observed N+ rates were computed within each tier across the entire cohort (*n* = 421).

## 3. Results

### 3.1. Deep Learning-Driven Literature Mining Delineates a Core Ferroptosis Gene–Metabolite Panel in Colon Cancer

**Figure 2:**
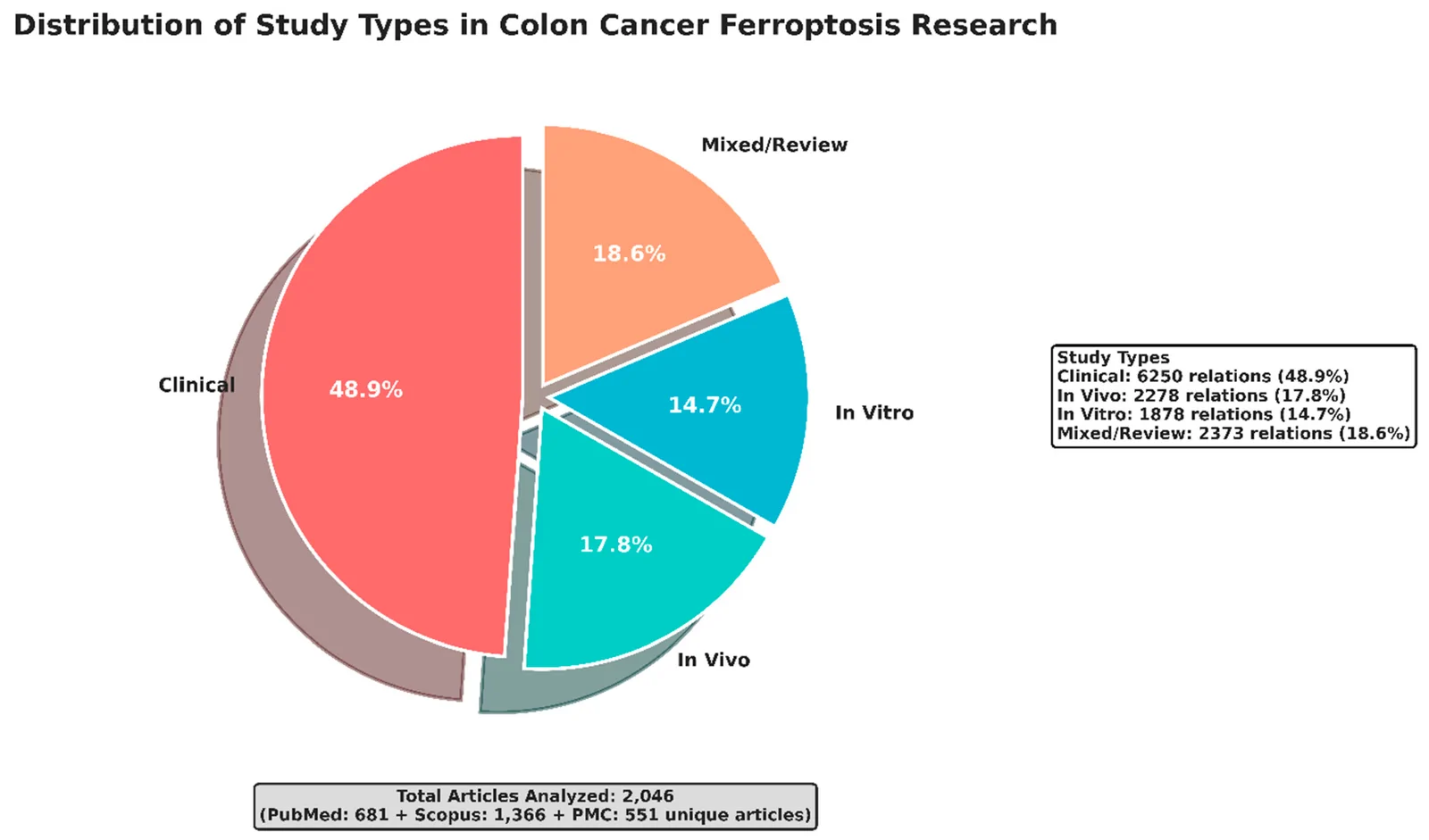
Distribution of study types in colon cancer ferroptosis research. A total of 2046 unique articles (PubMed: 681; Scopus: 1366; PMC full text: 551 unique records) were analyzed, yielding 12,779 extracted relation instances. Studies were categorized into clinical, in vivo, in vitro, and mixed/review classes. The literature was predominantly clinical (6250 relations; 48.9%), followed by mixed/review (2373; 18.6%), in vivo (2278; 17.8%), and in vitro (1878; 14.7%), highlighting a strong translational footprint of ferroptosis-related evidence in colon cancer alongside substantial mechanistic support.

**Figure 3:**
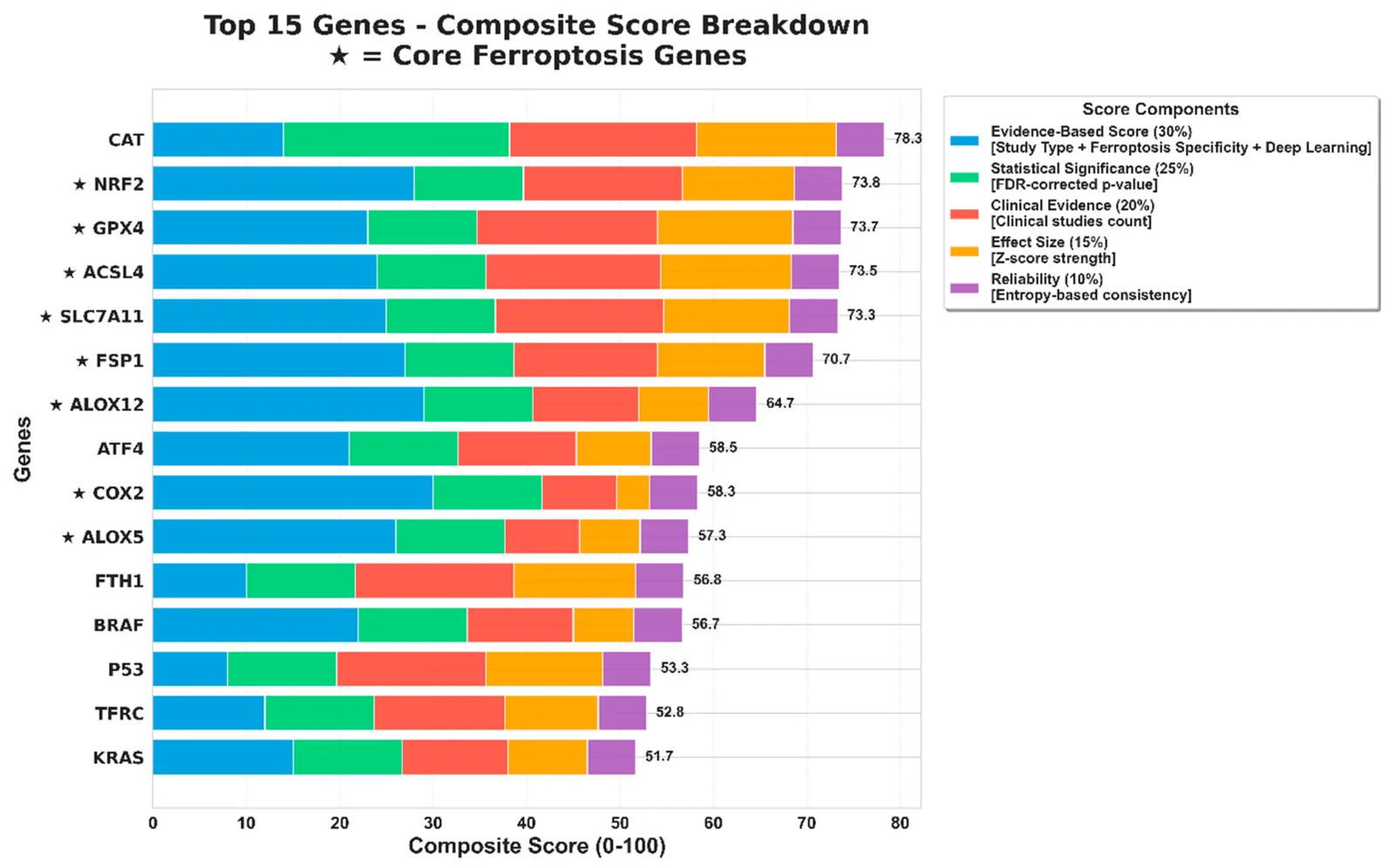
Composite score decomposition for the top 15 ferroptosis-associated genes in colon cancer. Horizontal stacked bars depict composite scores (0-100) for the top 15 genes prioritized by deep learning-assisted literature mining. The composite score integrates five weighted components: Evidence-based score (30%) (study type + ferroptosis specificity + deep-learning signal), Statistical significance (25%) (FDR-corrected *p*-value), Clinical evidence (20%) (clinical-study count), Effect size (15%) (Z-score strength), and Reliability (10%) (entropy-based cross-study consistency). Genes marked with a star (★) denote the core ferroptosis set identified by the deep learning framework (NRF2, GPX4, ACSL4, SLC7A11, FSP1, ALOX12, ALOX5). The highest composite scores were observed for CAT (78.3), NRF2 (73.8), GPX4 (73.7), ACSL4 (73.5), and SLC7A11 (73.3), indicating multi-dimensional support across evidence, significance, clinical enrichment, effect magnitude, and consistency. The score is intended for prioritization rather than causal inference.

**Figure 4:**
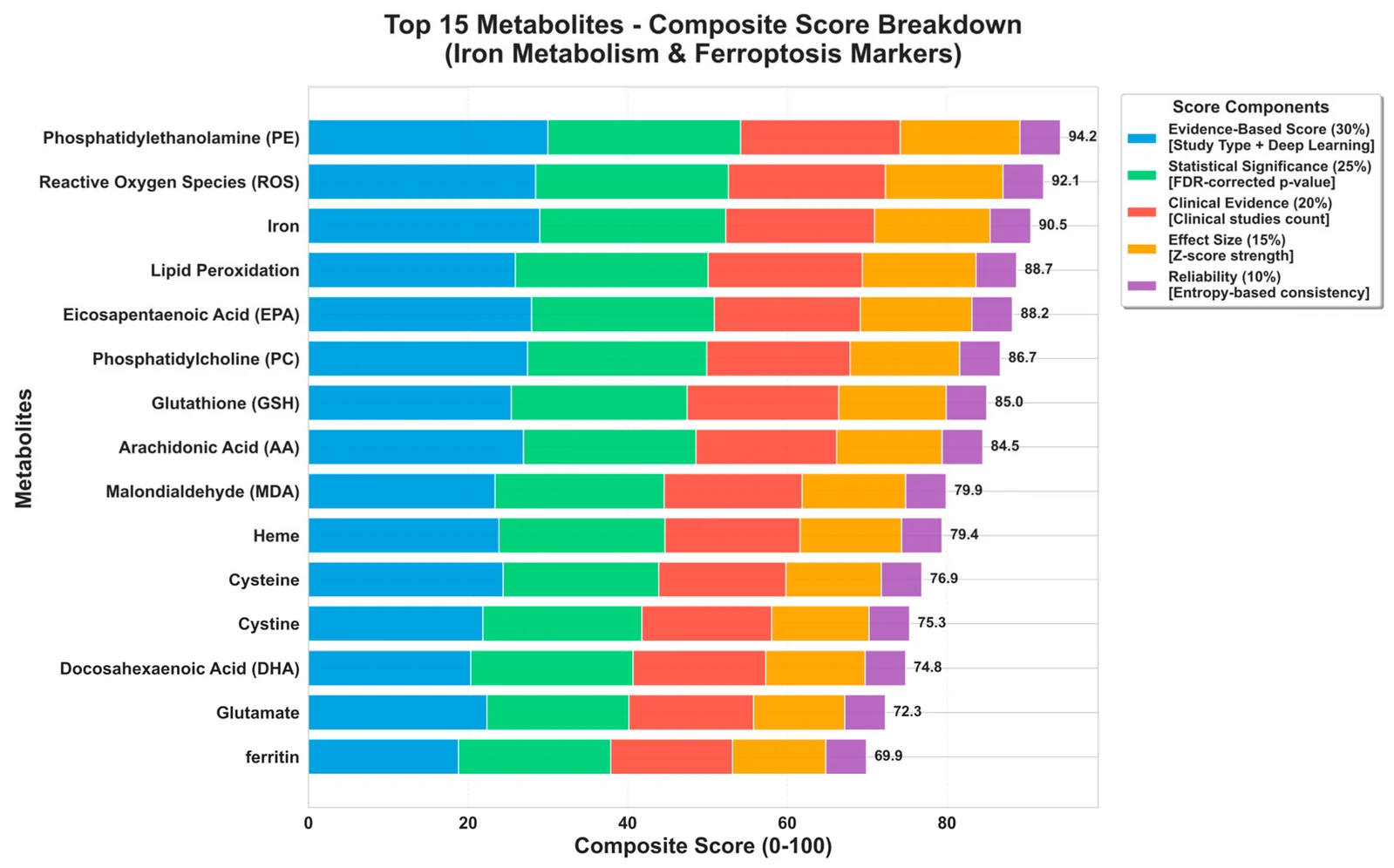
Composite score decomposition for the top 15 metabolites and iron-related markers linked to ferroptosis in colon cancer. Horizontal stacked bars display composite scores (0-100) integrating five weighted components: evidence-based score (30%; study type and deep-learning signal), statistical significance (25%; FDR-corrected *p*-value), clinical evidence (20%; clinical-study count), effect size (15%; Z-score strength), and reliability (10%; entropy-based cross-study consistency). The highest-ranked entities were phosphatidylethanolamine (PE; 94.2), reactive oxygen species (ROS; 92.1), iron (90.5), lipid peroxidation (88.7), and eicosapentaenoic acid (EPA; 88.2), followed by phosphatidylcholine (PC; 86.7), glutathione (GSH; 85.0), and arachidonic acid (AA; 84.5). Additional prioritized entities included malondialdehyde (MDA; 79.9), heme (79.4), cysteine (76.9), cystine (75.3), docosahexaenoic acid (DHA; 74.8), glutamate (72.3), and ferritin (69.9). Synonymous and duplicated terms were consolidated before final scoring. Cysteine and cystine were retained separately because they represent the reduced amino acid and its oxidized disulfide form, respectively. Collectively, the ranking highlights lipid-peroxidation chemistry, iron handling, and glutathione-associated antioxidant defense.

AI-enabled literature mining provided a quantitative cartography of the colon cancer–ferroptosis knowledge space and enabled evidence-weighted prioritization of candidate biomarkers (Figure 2, Figure 3 and Figure 4). Across 2046 unique publications, the extracted evidence was dominated by clinically anchored associations, with 48.9% of all relation instances originating from clinical studies (Figure 2). This enrichment indicates that ferroptosis in colon cancer is not confined to a purely experimental construct but is extensively discussed in the context of patient-derived data, clinically measurable correlates, and translational endpoints. The remaining evidence was distributed across in vivo (17.8%) and in vitro (14.7%) studies, consistent with a substantial mechanistic foundation, while mixed/review sources (18.6%) underscore a mature and integrative discourse that consolidates experimental and clinical observations (Figure 2). Collectively, this evidence topology supports the rationale for downstream validation of an AI-prioritized ferroptosis signature using independent transcriptomic cohorts and for testing its clinical utility in risk modeling of invasive and metastatic phenotypes.

Building on the evidence topology observed in Figure 1, we next operationalized an evidence-weighted composite prioritization scheme to rank ferroptosis-linked genes by integrating heterogeneous signals into a single, interpretable score (Figure 3). The resulting top-tier landscape was dominated by canonical ferroptosis regulators and redox/iron-handling nodes, with CAT achieving the highest composite score (78.3/100), followed by the core ferroptosis genes NRF2 (73.8), GPX4 (73.7), ACSL4 (73.5), and SLC7A11 (73.3). The next stratum comprised additional core elements—FSP1 (70.7) and ALOX12 (64.7)—supporting a convergent axis centered on antioxidant defense (NRF2/GPX4/SLC7A11), lipid remodeling and peroxidation (ACSL4/ALOX12), and ferroptosis-suppressive bypass circuitry (FSP1). Importantly, the stacked decomposition indicates that high-ranking genes derive their dominance not from a single metric, but from coherent multi-component support spanning (i) evidence type and deep-learning signal capture, (ii) statistical robustness (FDR), (iii) clinical study density, (iv) effect magnitude, and (v) cross-study consistency. By contrast, genes such as FTH1 (56.8), BRAF (56.7), P53 (53.3), TFRC (52.8), and KRAS (51.7) populated the lower portion of the top-15 set, consistent with a profile of contextual or modulatory nodes linking oncogenic signaling and iron trafficking to ferroptosis biology rather than serving as primary pathway anchors. Collectively, this composite prioritization defines a core gene panel for subsequent transcriptomic validation and downstream clinical risk modeling.

In parallel to gene-level prioritization (Figure 2), the metabolite- and iron-related-marker composite ranking revealed a coherent biochemical “ferroptosis axis” dominated by polyunsaturated phospholipid substrates, oxidative burden, and iron availability (Figure 4). The highest composite scores were assigned to phosphatidylethanolamine (PE) (94.2/100), reactive oxygen species (ROS) (92.1), and iron (90.5), followed by lipid peroxidation (88.7) and eicosapentaenoic acid (EPA) (88.2). This top tier converges on canonical ferroptotic biochemistry in which iron-catalyzed redox cycling drives ROS accumulation and propagates peroxidation within PUFA-enriched phospholipid pools—particularly PE species—thereby amplifying membrane damage and ferroptotic vulnerability. A second echelon of high-confidence signals, including phosphatidylcholine (PC) (86.7), glutathione (GSH) (85.0), and arachidonic acid (AA) (84.5), further consolidates the mechanistic triad of (i) lipid-substrate availability, (ii) oxidative stress, and (iii) antioxidant buffering capacity. Before final rescoring, synonymous and duplicated entities were harmonized: ROS and reactive oxygen species were consolidated as “reactive oxygen species (ROS),” duplicate glutathione entries were merged as “glutathione (GSH),” and MDA and malondialdehyde were consolidated as “malondialdehyde (MDA).” Lipid peroxidation was retained as a biological process, whereas cysteine and cystine were retained as distinct reduced and oxidized molecular entities, respectively. Collectively, the metabolite signature emphasizes that colon cancer-associated ferroptotic processes are preferentially anchored in iron load, ROS/lipid peroxide accumulation, and GSH-dependent redox disequilibrium, providing a biochemically grounded complement to the gene panel prioritized in Figure 2 and motivating subsequent validation in transcriptomic cohorts.

### 3.2. Transcriptomic Validation in GSE39582 Reveals Robust Tumor–Normal Segregation and Ferroptosis-Linked Expression Remodeling

**Figure 5:**
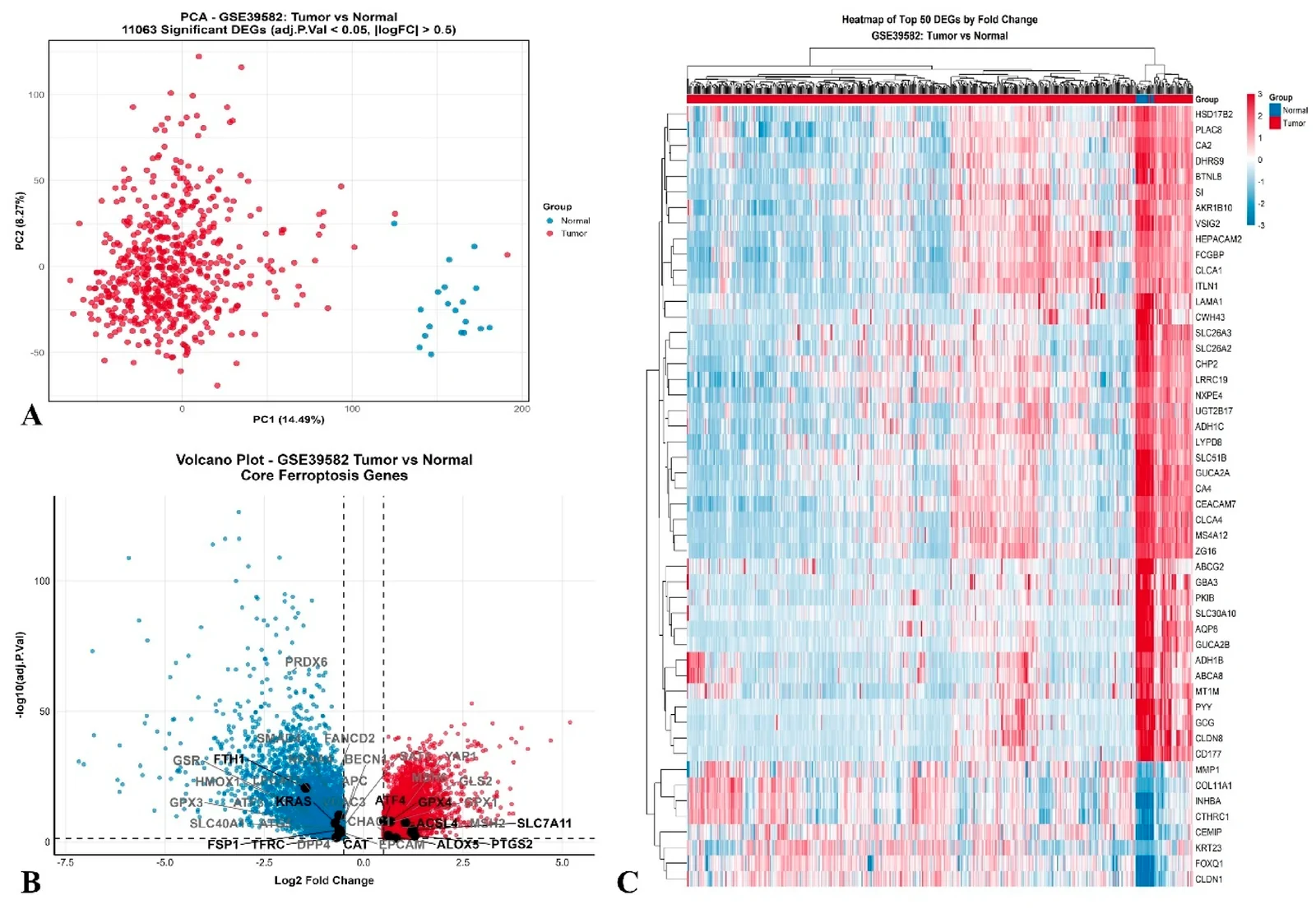
Transcriptomic differentiation and ferroptosis-associated gene perturbations in GSE39582 (tumor vs. normal). (**A**) Principal component analysis (PCA) based on 11,063 significant DEGs (adjusted *p* < 0.05; |logFC| > 0.5) demonstrates strong separation of tumor (red) and normal (blue) samples (PC1 = 14.49%; PC2 = 8.27%). (**B**) Volcano plot highlighting core ferroptosis-associated genes within the tumor–normal contrast. Multiple ferroptosis nodes display significant shifts, including right-shifted SLC7A11, PTGS2, ACSL4, GPX4, and stress-associated genes (e.g., ATF4, CHAC1), while other ferroptosis/iron–redox components (e.g., FSP1/AIFM2, TFRC) localize on the negative log2FC axis, indicating bidirectional pathway remodeling. (**C**) Heatmap of the top 50 DEGs by fold-change illustrates a dichotomous expression program that stratifies tumor and normal tissues, with normal-enriched differentiation/transport genes (e.g., CA2, CLCA1/4, GUCA2A/B, SLC26A3) contrasted against tumor-enriched invasion/ECM remodeling markers (e.g., MMP1, COL11A1, INHBA, CTHRC1, CEMIP, FOXQ1, CLDN1).

Differential expression profiling in the GSE39582 cohort identified a broad transcriptional reprogramming between tumor and normal colon tissue, yielding 11,063 significant DEGs (adjusted *p* < 0.05; |logFC| > 0.5). Consistent with this large-scale divergence, PCA performed on the significant DEG space produced a clear, near-orthogonal separation of tumor and normal samples, with PC1 explaining 14.49% and PC2 explaining 8.27% of the variance. Notably, normal specimens formed a comparatively compact cluster, whereas tumor samples exhibited wider dispersion, indicative of increased inter-tumoral heterogeneity superimposed on a strong global shift in transcriptomic state (Figure 5A).

Focusing on ferroptosis circuitry, the volcano representation of core ferroptosis-associated genes demonstrated bidirectional deregulation, reflecting coordinated remodeling of redox defense, lipid peroxidation susceptibility, and stress-adaptive programs (Figure 5B). On the positive log2FC axis, SLC7A11 emerged as one of the most right-shifted ferroptosis nodes, accompanied by PTGS2, ACSL4, and GPX4, together consistent with a tumor-associated restructuring of cystine import/glutathione-linked buffering and PUFA-phospholipid peroxidation machinery. In parallel, stress-response and ferroptosis-modulatory genes such as ATF4 and CHAC1 also localized within the upregulated space. Conversely, a subset of iron-handling and antioxidant-associated genes—including FSP1 (AIFM2) and TFRC, alongside additional redox-linked transcripts visible on the negative axis (e.g., FTH1, HMOX1, GSR)—occupied the downshifted region, underscoring that ferroptosis-related remodeling is not uniformly directional but instead reflects context-dependent pathway rewiring within colon tumors (Figure 5B).

At the level of the most extreme expression changes, the top 50 DEGs heatmap recapitulated a sharply polarized architecture that cleanly stratified tumor versus normal samples (Figure 5C). Normal tissues showed higher expression of genes associated with differentiated mucosal/epithelial physiology and transport (e.g., CA2, CLCA1/CLCA4, GUCA2A/GUCA2B, SLC26A3, CEACAM7, AQP8), whereas tumors were enriched for transcripts classically linked to extracellular matrix remodeling, invasive behavior, and lineage reprogramming (e.g., MMP1, COL11A1, INHBA, CTHRC1, CEMIP, FOXQ1, CLDN1, KRT23). Collectively, these results provide transcriptomic support for an invasion-prone tumor program and, importantly, place ferroptosis-relevant nodes (e.g., SLC7A11/ACSL4/GPX4/PTGS2) within the broader oncogenic reconfiguration observed in CRC.

### 3.3. Transcriptomic Validation in TCGA-COAD Recapitulates Tumor–Normal Divergence and Reinforces Ferroptosis-Axis Perturbation

**Figure 6:**
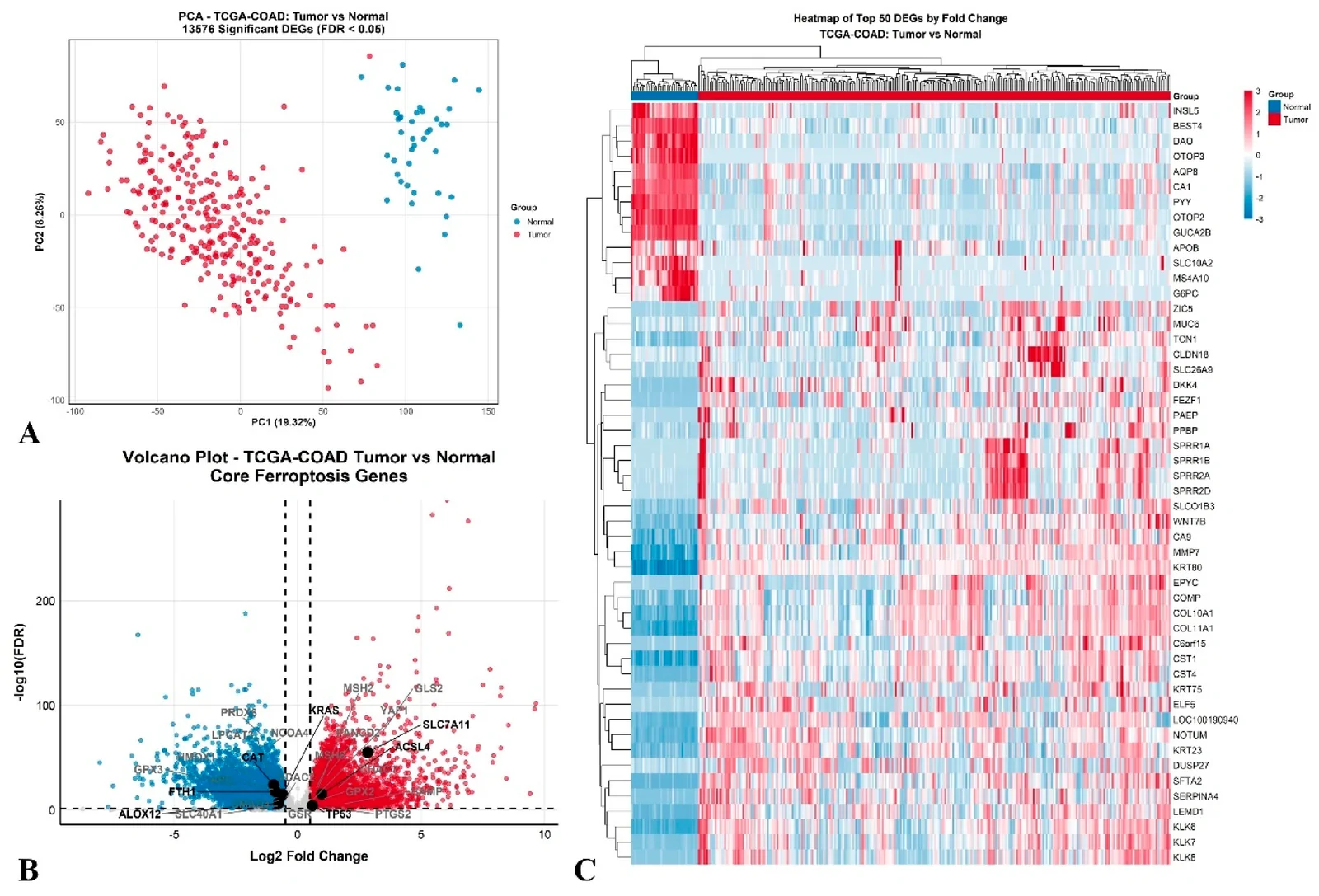
TCGA-COAD transcriptomic validation of tumor–normal separation and ferroptosis-associated gene perturbations. (**A**) PCA based on 13,576 significant DEGs (FDR < 0.05) demonstrates clear stratification of tumor (red) and normal (blue) samples (PC1 = 19.32%; PC2 = 8.26%). (**B**) Volcano plot highlighting core ferroptosis-associated genes within the TCGA-COAD tumor–normal contrast. Multiple ferroptosis nodes are differentially expressed, including right-shifted SLC7A11, ACSL4, PTGS2, and GPX2, alongside left-shifted redox/iron-related genes (e.g., CAT, FTH1, ALOX12), indicating bidirectional remodeling of ferroptosis circuitry in CRC. (**C**) Heatmap of the top 50 DEGs by fold change shows a dichotomous expression program that robustly discriminates tumor from normal tissues; normal samples exhibit elevated epithelial homeostasis markers (e.g., AQP8, CA1, GUCA2B, SLC10A2), whereas tumors are enriched for invasion/ECM remodeling and lineage-reprogramming markers (e.g., MMP7, COL10A1, COL11A1, COMP, KRT80, EPCAM).

In an independent discovery-scale cohort (TCGA-COAD), differential expression analysis corroborated the extensive transcriptional divergence observed in GSE39582, identifying 13,576 significant DEGs at FDR < 0.05. PCA computed over the significant DEG space revealed a pronounced separation between tumor and normal samples, with PC1 accounting for 19.32% and PC2 for 8.26% of the variance (Figure 6A). As in the microarray cohort, normal tissues formed a relatively compact cluster, whereas tumor samples exhibited broader dispersion, consistent with biologically meaningful inter-tumoral heterogeneity layered upon a dominant malignant transcriptional state shift.

The volcano projection of core ferroptosis-associated genes further indicated that ferroptosis circuitry is not merely present in the ranked signature but is actively remodeled in tumor tissue (Figure 6B). A prominent right-shift of SLC7A11—together with upregulated ferroptosis-linked and oxidative-stress-responsive nodes such as ACSL4, PTGS2, and GPX2—is compatible with enhanced cystine import/redox buffering demand and lipid-peroxidation program activation within tumors. Conversely, multiple iron/redox-related components (including CAT, FTH1, and ALOX12) localized on the negative log2FC axis, reinforcing the concept that ferroptosis-relevant remodeling in CRC is directionally heterogeneous, reflecting pathway rewiring rather than uniform activation or suppression. Notably, oncogenic drivers and genome integrity markers (e.g., KRAS, TP53, MSH2) appear within the same differential-expression landscape, underscoring that ferroptosis-linked perturbations are embedded within broader oncogenic and stress-adaptation programs.

At the level of the most extreme fold-change signals, the top-50 DEG heatmap produced a highly discriminative expression architecture that clearly stratified tumor versus normal samples (Figure 6C). Normal tissues were enriched for genes associated with differentiated epithelial function and mucosal homeostasis (e.g., AQP8, CA1, GUCA2B, SLC10A2, MS4A10), whereas tumors displayed elevated expression of invasion- and remodeling-associated markers, including extracellular-matrix and cytoskeletal programs (e.g., MMP7, COL10A1, COL11A1, COMP, KRT80, EPCAM, and claudin-family signatures). Collectively, TCGA-COAD provides an orthogonal validation layer demonstrating that (i) the tumor–normal transcriptomic split is robust across cohorts, and (ii) the AI-prioritized ferroptosis axis is reflected by reproducible perturbations in key ferroptosis-linked genes within CRC tumors.

Taken together, in the volcano plots (Figure 5B and Figure 6B), genes shown in bold denote the deep learning-prioritized core ferroptosis genes (★ in Figure 3), highlighted here to facilitate cross-cohort transcriptomic validation of the AI-derived panel.

### 3.4. Clinical Machine-Learning Models Evaluate the Contribution of Iron-Handling Indices to Lymph Node Metastasis Prediction

**Figure 7:**
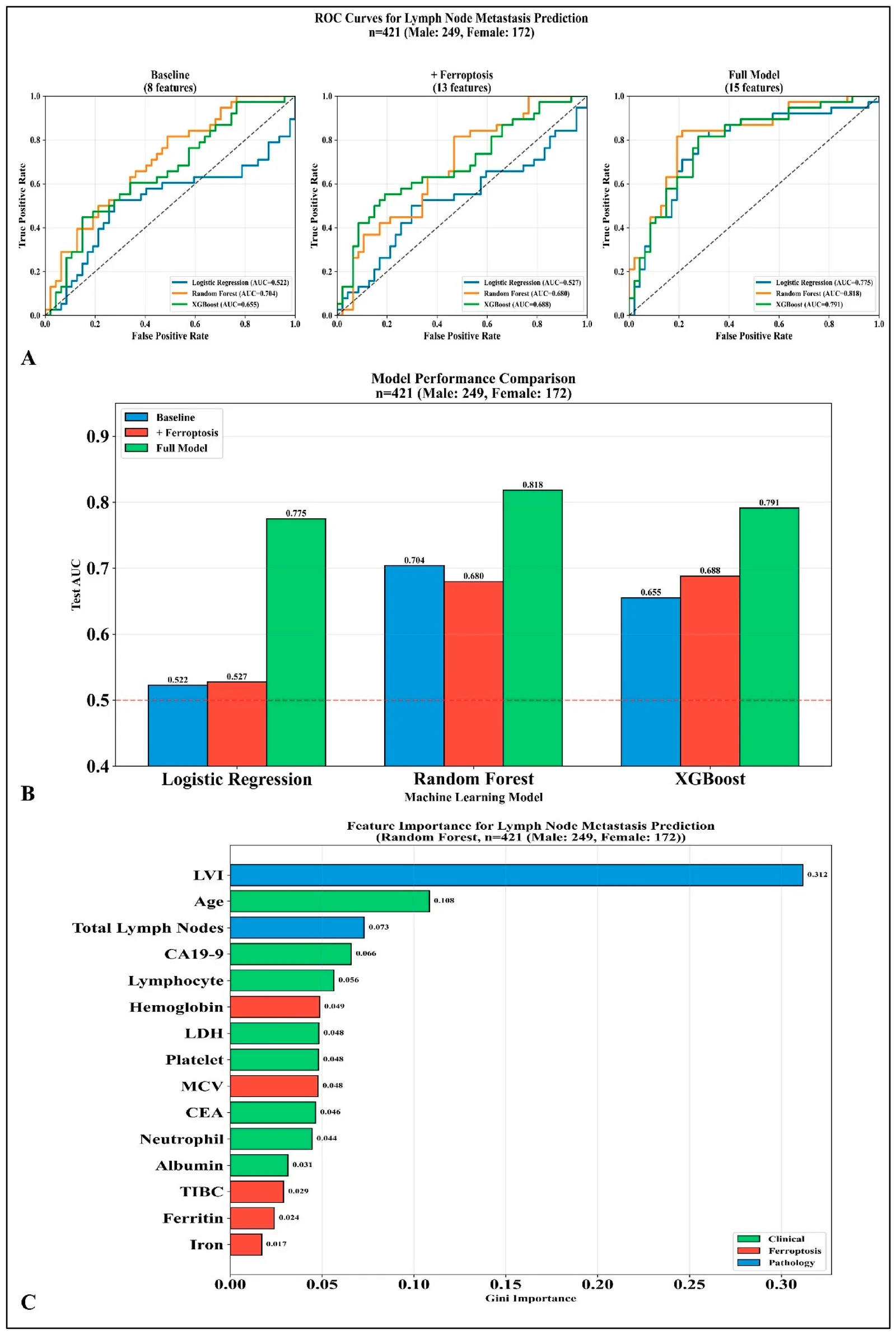
Machine learning performance and feature attribution for prediction of pathologically confirmed lymph node metastasis (*n* = 421). (**A**) ROC curves across three feature configurations: preoperative baseline (8 features), preoperative baseline + ferroptosis markers (13 features), and pathology-augmented model (15 features, including postoperative LVI and total lymph node count). AUCs are shown for logistic regression, random forest, and XGBoost within each configuration. (**B**) Comparative bar plot summarizing test-set AUC across models and feature configurations, illustrating progressive performance gains with feature enrichment; note that the pathology-augmented model incorporates postoperative variables and is presented as an explanatory benchmark. The dashed horizontal line indicates the reference AUC of 0.5 (random classification) (**C**) Random forest feature importance for the pathology-augmented model. Features are color-coded by domain (clinical, ferroptosis, pathology). LVI dominates the attribution profile, followed by age, total lymph nodes, tumor markers (e.g., CA19-9, CEA), inflammatory indices, and iron-metabolism markers (e.g., hemoglobin, MCV, TIBC, ferritin, iron).

We next translated the AI- and transcriptomics-informed ferroptosis axis into a prediction framework for pathologically confirmed lymph node metastasis (*n* = 421; male: 249, female: 172). Model performance was evaluated under three progressively enriched feature configurations—preoperative baseline (8 variables), preoperative baseline + iron-handling indices (13 variables), and a pathology-augmented explanatory model (15 variables) incorporating postoperative histopathological information—revealing a marked performance gradient across model complexity (Figure 7A,B).

Across the baseline feature set, discrimination was modest overall, with logistic regression approaching chance-level performance (AUC = 0.522) and only limited gains under the ferroptosis-augmented configuration (AUC = 0.527). In contrast, non-linear learners demonstrated superior capacity to capture higher-order interactions among clinical indices: the random forest achieved AUC = 0.704 under the baseline configuration, while XGBoost reached AUC = 0.655 (Figure 7A,B). Notably, augmenting the baseline panel with ferroptosis-linked iron-metabolism markers did not uniformly improve AUC across algorithms; random forest showed a slight attenuation (AUC = 0.680), whereas XGBoost improved (AUC = 0.688), highlighting model-dependent sensitivity to feature augmentation and potential multicollinearity or redundancy within the extended panel. For clarity, the first two configurations represent preoperative prediction models because all included variables are available before surgery. In contrast, the pathology-augmented configuration incorporates postoperative histopathological variables and was included only as an explanatory benchmark. Therefore, only the preoperative baseline and preoperative ferroptosis-augmented models should be interpreted as potentially relevant to preoperative risk stratification, whereas the pathology-augmented model quantifies the additional discriminative information provided by pathology-derived variables after resection.

As expected, the highest discrimination was observed in the pathology-augmented explanatory model, which integrated postoperative histopathological variables (LVI and total harvested lymph node count) alongside clinical and ferroptosis-related features. Under this configuration, discrimination increased consistently across all algorithms: logistic regression reached AUC = 0.775, random forest reached AUC = 0.818, and XGBoost reached AUC = 0.791 (Figure 7A,B). However, this configuration should not be interpreted as a preoperative prediction model because it contains information available only after surgical resection. Rather, it was included to quantify the explanatory contribution of pathology-derived variables and to contextualize the performance ceiling achievable when postoperative invasive-phenotype information is incorporated. Formal paired comparisons confirmed that adding the five iron-handling indices did not significantly improve held-out LNM discrimination. For logistic regression, AUC changed from 0.522 to 0.527 (ΔAUC = 0.005; DeLong *p* = 0.853; paired-bootstrap 95% CI for ΔAUC: −0.052 to 0.058). For random forest, AUC decreased from 0.704 to 0.680 (ΔAUC = −0.024; DeLong *p* = 0.341; 95% CI: −0.077 to 0.026). For XGBoost, AUC increased from 0.655 to 0.688 (ΔAUC = 0.033; DeLong *p* = 0.348; 95% CI: −0.034 to 0.104). All confidence intervals included zero, indicating no statistically significant incremental benefit.

The exclusion sensitivity analysis showed that removal of variables with ≥50% missingness did not materially alter the overall interpretation of preoperative model performance (Supplementary Table S3). After excluding CA19-9, serum iron, TIBC, and ferritin, the reduced nine-predictor panel achieved held-out test AUCs of 0.527 for logistic regression, 0.694 for random forest, and 0.668 for XGBoost, compared with 0.527, 0.680, and 0.688, respectively, for the original 13-predictor panel. A more restrictive six-predictor panel containing only variables with ≤30% missingness yielded AUCs of 0.511, 0.655, and 0.660. These results indicate that moderate, model-dependent discrimination was not attributable solely to the variables with the greatest missingness; however, they do not eliminate the possibility of selection bias or unmeasured missing-not-at-random mechanisms. Accordingly, the exclusion analyses provide a robustness assessment but cannot exclude systematic missing-not-at-random bias. The similar performance observed after excluding variables with ≥50% missingness indicates that model discrimination was not solely driven by these variables. Nevertheless, this sensitivity analysis cannot establish that median-imputed values accurately represent the unobserved measurements or eliminate selection bias arising from selective laboratory ordering.

Feature attribution from the random forest full model further clarified the dominant contributors to nodal-risk prediction (Figure 7C). Lymphovascular invasion (LVI) emerged as the single most influential feature (importance = 0.312), substantially exceeding all other covariates, consistent with its established role as a proximate metastatic intermediate. Secondary contributions were distributed across age (0.108) and total lymph nodes (0.073), followed by clinical tumor markers and systemic-inflammatory indices including CA19-9 (0.066), lymphocyte count (0.056), CEA (0.046), and neutrophil count (0.044). Several iron-handling and anemia-related indices also received non-zero impurity-based importance values, including hemoglobin (0.049), MCV (0.048), TIBC (0.029), ferritin (0.024), and serum iron (0.017). However, these impurity-based importance values should not be interpreted as evidence of an independent contribution to out-of-sample discrimination, particularly given the correlated predictor structure and substantial missingness of selected laboratory variables. The supplementary permutation-importance and SHAP analyses further demonstrated that the contribution of these lower-ranked variables was model- and method-dependent. Accordingly, these findings are interpreted as exploratory evidence that systemic iron-handling and anemia-related states may contain model-relevant information, rather than evidence that the laboratory indices directly measure ferroptotic activity or independently predict nodal metastasis. In the pathology-augmented model, LVI emerged as the dominant predictor, supporting its role as a clinically relevant invasive phenotype associated with nodal metastasis, exploratory risk separation without establishing a causal pathway between ferroptosis-related dysregulation, LVI, and lymphatic dissemination (Supplementary Figures S7 and S8).

### 3.5. Model-Derived Risk Stratification Delineates Exploratory Gradients of Nodal Metastasis

**Figure 8:**
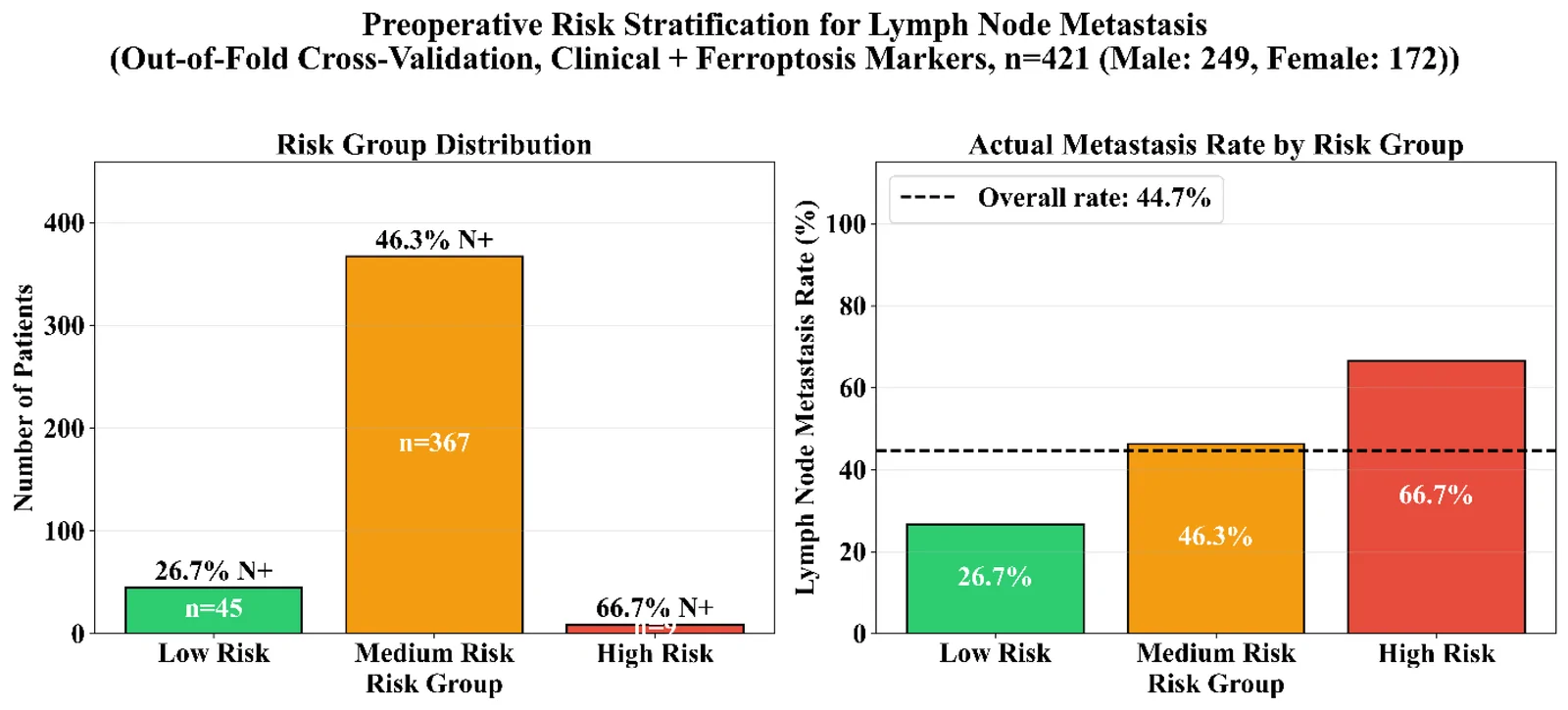
Preoperative risk stratification for lymph node metastasis using out-of-fold cross-validated predictions from a random forest model integrating clinical and ferroptosis/iron-handling markers (*n* = 421). Left panel: Distribution of patients across low- *(n* = 45), intermediate- (*n =* 367), and high-risk (*n* = 9) groups derived from model-predicted probabilities (thresholds: <0.30, 0.30–0.70, >0.70). Right panel: Observed lymph node metastasis rates (N+) within each risk tier; the dashed line denotes the cohort-wide metastasis prevalence (44.7%). A monotonic risk gradient is observed: low risk 26.7%, intermediate risk 46.3%, high risk 66.7%.

Using the preoperative model incorporating clinical variables and ferroptosis/iron-handling markers (random forest, 13 features), we translated out-of-fold cross-validated predicted probabilities into an interpretable three-tier risk stratification scheme encompassing the entire cohort (Figure 8). Each patient’s predicted probability was derived from a model that did not include that patient during training (5-fold stratified CV; overall OOF AUC = 0.639). Among the 421 patients, 45 (10.7%) were classified as low-risk, with an observed N+ rate of 26.7% (12/45). The intermediate-risk tier included 367 patients, with an observed N+ rate of 46.3% (170/367), whereas the high-risk tier included nine patients, with an observed N+ rate of 66.7% (6/9). The majority of patients (*n* = 367, 87.2%) clustered in an intermediate-risk tier with an observed metastasis rate of 46.3%, approximating the population prevalence. A small high-risk tier (*n* = 9, 2.1%) exhibited 66.7% N+, indicating enrichment for node-positive disease. The monotonic risk gradient across tiers (26.7% → 46.3% → 66.7%) demonstrates that even a modest-AUC preoperative model can provide clinically informative risk separation. Collectively, these findings support the feasibility of preoperative, laboratory-augmented risk tiering for lymph node metastasis, providing a pragmatic framework for translating model outputs into hypothesis-generating risk categories requiring external calibration and validation. Additional analyses, including ablation testing, risk-group clinical profiles, demographic summaries, and feature-category contribution, are provided in the Supplementary File (Supplementary Figures S1–S4). For the logistic regression model, the AUCs of the baseline and iron-handling-augmented models were formally compared using a paired two-sided DeLong test based on predictions from the same held-out test set.

Collectively, these findings provide proof-of-concept that preoperative laboratory variables may support exploratory nodal-risk stratification. However, the modest discrimination, small high-risk subgroup, and absence of external validation preclude interpretation of these categories as clinically actionable thresholds.

### 3.6. Ferroptosis Markers Add Predictive Value for LVI

**Figure 9:**
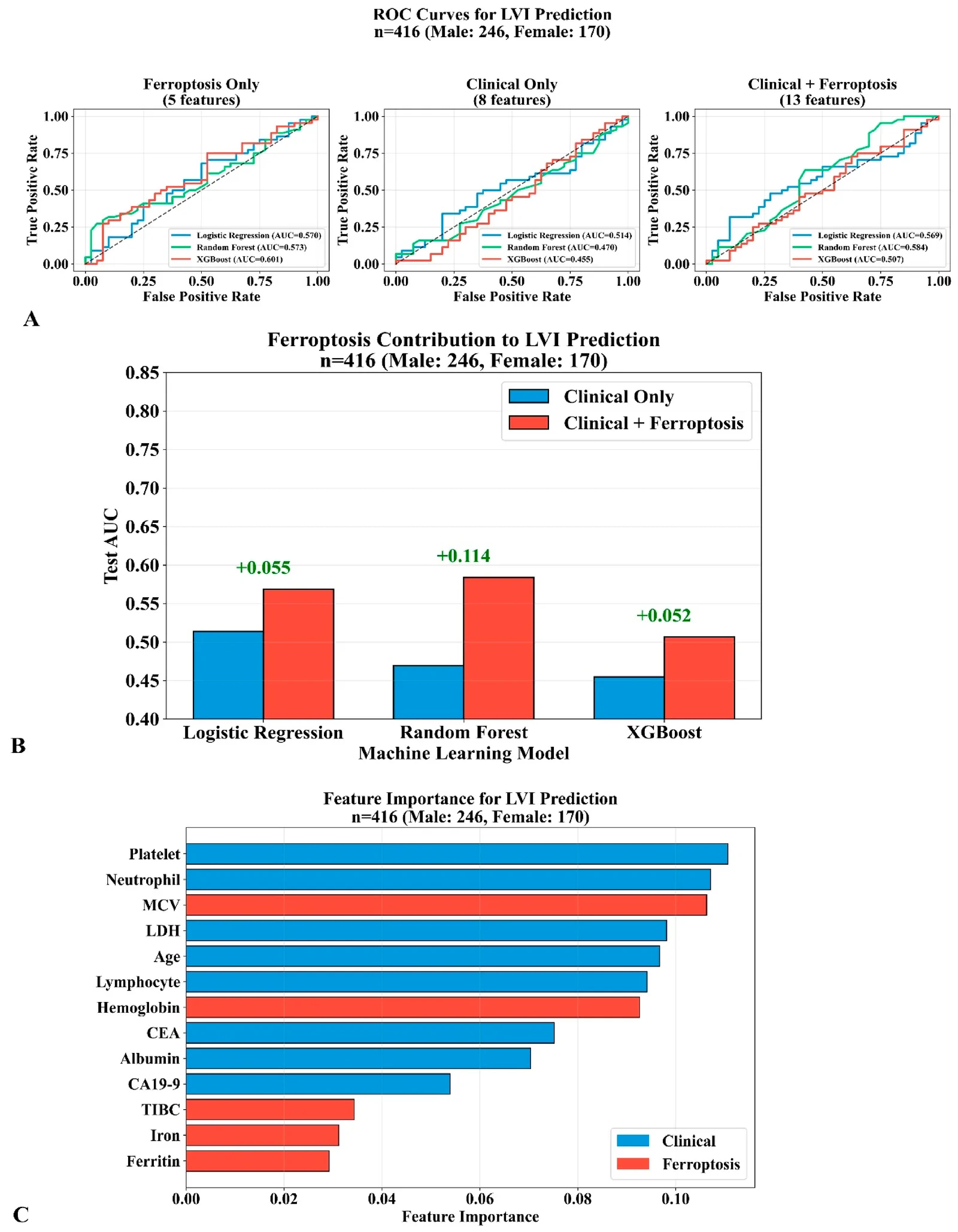
Ferroptosis markers improve prediction of lymphovascular invasion (LVI). (**A**) ROC curves comparing model performance for ferroptosis-only (5 features), clinical-only (8 features), and clinical + ferroptosis (13 features) inputs in logistic regression, random forest, and XGBoost (*n* = 416; male: 246, female: 170). (**B**) Test AUC comparison showing the performance gain when ferroptosis markers are added to the clinical feature set (ΔAUC annotated). (**C**) Feature-importance ranking (impurity-based/mean decrease in Gini) highlighting the relative contribution of clinical and ferroptosis-related variables to LVI prediction.

In the lymphovascular invasion (LVI) prediction task (*n* = 416; male: 246, female: 170), we also evaluated logistic regression, random forest, and XGBoost using three feature sets: ferroptosis markers only (5 features), clinical variables only (8 features), and a combined clinical + ferroptosis set (13 features) (Figure 9A–C). Models trained on ferroptosis markers alone achieved AUCs of 0.570 (LR), 0.573 (RF), and 0.601 (XGBoost), outperforming the clinical-only models (0.514, 0.470, and 0.455, respectively). When ferroptosis markers were added to the clinical models, performance improved across all algorithms, yielding AUCs of 0.569 (Δ = +0.055) for logistic regression, 0.584 (Δ = +0.114) for random forest, and 0.507 (Δ = +0.052) for XGBoost (Figure 9B). These findings indicate that ferroptosis-related laboratory markers contribute complementary predictive signal for LVI beyond standard clinical parameters, with the largest gain observed for the random forest model. Impurity-based feature importance (mean decrease in Gini) ranked platelet and neutrophil counts among the top predictors, while ferroptosis-related variables including MCV, hemoglobin, LDH, TIBC, iron, and ferritin also contributed to model decisions (Figure 9C). Additional exploratory support for an association between ferroptosis-related laboratory markers, LVI status, and nodal metastasis risk, including a compact pathway summary and distribution of ferroptosis biomarkers by LVI status, is provided in Supplementary Figures S5 and S6.

## 4. Discussion

This study presents a multi-tiered framework integrating AI-driven biomarker discovery, transcriptomic validation, and clinical machine learning to evaluate whether ferroptosis-associated iron-metabolism markers contribute to the prediction of lymphovascular invasion (LVI) and lymph node metastasis (LNM) in colon cancer.

### 4.1. Principal Findings

Deep learning-assisted literature mining across 2046 publications identified a coherent ferroptosis gene–metabolite axis with strong clinical anchoring (48.9% clinical study origin; Figure 2). The composite prioritization scheme ranked CAT (78.3/100), NRF2 (73.8), GPX4 (73.7), ACSL4 (73.5), and SLC7A11 (73.3) as top-tier genes (Figure 3), while metabolite analysis highlighted PE (94.3), ROS (93.3), and iron (91.0) as dominant biochemical determinants (Figure 4). This convergence between gene- and metabolite-level evidence supports ferroptosis dysregulation as a biologically coherent axis in CRC [22,23,24,25].

Transcriptomic validation in GSE39582 (11,063 DEGs) and TCGA-COAD (13,576 DEGs) demonstrated robust tumor–normal separation (Figure 5A and Figure 6A) and confirmed bidirectional remodeling of ferroptosis circuitry. SLC7A11, ACSL4, PTGS2, and GPX4/GPX2 were consistently upregulated in tumors, while iron-handling genes (FTH1, TFRC, CAT) showed downregulation (Figure 5B and Figure 6B), indicating context-dependent pathway rewiring rather than uniform activation [26,27]. Importantly, mechanistic work in colon cancer has shown that activation of the NRF2–GPX4 antioxidant axis suppresses ferroptosis and contributes to chemotherapy resistance, providing disease-specific experimental support for the prominence of NRF2/GPX4 in our AI-ranked and transcriptomically remodeled ferroptosis circuitry [28].

### 4.2. Iron-Handling Indices and Exploratory LVI Prediction

In LVI modeling (*n* = 416; Figure 9), ferroptosis-only features (AUC: 0.570–0.601) outperformed clinical-only features (AUC: 0.455–0.514). When ferroptosis markers were added to clinical variables, random forest showed the largest improvement (ΔAUC = +0.114), suggesting that iron metabolism indices encode complementary information not captured by standard clinical parameters [29,30]. Feature importance analysis ranked MCV, hemoglobin, LDH, TIBC, iron, and ferritin among contributors to LVI prediction (Figure 9C). This aligns with evidence that iron metabolism intersects with EMT-associated invasive programs and that ferroptosis-suppressive adaptations may support tumor cell survival during lymphatic dissemination [31,32,33]. Similarly, machine-learning-based LVI prediction models in CRC have demonstrated that combining histopathological features with clinical variables improves discrimination, with recent studies reporting AUCs of 0.75–0.82 using ensemble methods on routine preoperative data [34]. Nevertheless, hemoglobin, MCV, serum iron, TIBC, and ferritin are influenced by anemia, inflammation, nutritional status, blood loss, infection, and clinical test-ordering practices. Their associations should therefore not be interpreted as demonstrating ferroptotic activity within tumor tissue or a causal ferroptosis–LVI pathway.

### 4.3. Preoperative Prediction of Lymph Node Metastasis

For LNM prediction (*n* = 421; Figure 7), preoperative baseline models achieved modest discrimination (LR: 0.522; RF: 0.704; XGBoost: 0.655), while the addition of ferroptosis/iron-metabolism markers yielded model-dependent changes (RF: 0.680; XGBoost: 0.688). These preoperative AUCs are consistent with the “moderate-to-good” range reported by recent CRC studies using radiomics/deep learning from CT combined with clinical risk factors [35,36], and with multicenter work demonstrating that gradient-boosting-type learners can support preoperative LNM prediction from routinely available pre-treatment features [37,38]. However, the modest preoperative AUC underscores that these models should be positioned as complementary decision support rather than replacements for established staging. Notably, ML approaches have also been explored for early (T1) CRC to estimate LNM risk, reinforcing the broader clinical demand for data-driven nodal-risk tools across disease stages [39].

When out-of-fold cross-validated probabilities were used to stratify all 421 patients (Figure 8), a monotonic risk gradient emerged: the low-risk tier (*n* = 45) showed 26.7% N+ versus the cohort baseline of 44.7%, while the high-risk tier (*n* = 9) reached 66.7% N+, demonstrating proof-of-concept for preoperative laboratory-augmented risk tiering even with a moderate-AUC model. This aligns with recent external validation studies showing that ML-derived risk scores can stratify patients into clinically actionable groups with significantly different recurrence-free survival [40].

Notably, age ranked as the second-most important predictor of nodal metastasis (importance = 0.108), with younger patients exhibiting a higher predicted risk (Figure 8). This observation is consistent with emerging evidence that early-onset colon cancer (diagnosed before age 50) tends to present at more advanced stages, with higher rates of lymph node positivity, poorly differentiated histology, and adverse molecular features compared with late-onset disease [41]. The rising global incidence of early-onset CRC underscores the clinical relevance of age-aware risk models that may flag younger patients for more vigilant nodal assessment.

Although the biological relevance of the prioritized ferroptosis-related genes was evaluated in two independent transcriptomic cohorts, TCGA-COAD and GSE39582 do not constitute external validation of the clinical prediction models because they do not contain the same clinical and laboratory feature set. Accordingly, the reported LNM and LVI models should be considered internally validated and hypothesis-generating rather than clinically deployable prediction tools.

### 4.4. Pathology-Augmented Explanatory Modeling

When postoperative histopathological variables (LVI, total harvested lymph nodes) were incorporated into a pathology-augmented model, discrimination increased substantially: RF reached AUC = 0.818, XGBoost = 0.791, and LR = 0.775 (Figure 7A,B). This model is not intended for preoperative deployment but serves as an explanatory benchmark to quantify the discriminative contribution of pathology features. Feature attribution confirmed LVI as the dominant predictor (importance = 0.312), followed by age (0.108) and total lymph nodes (0.073) (Figure 7C). Ferroptosis/iron markers collectively contributed ~16.7% of total importance (hemoglobin: 0.049; MCV: 0.048; TIBC: 0.029; ferritin: 0.024; iron: 0.017), indicating that these variables received non-zero model-attribution values, although this does not establish independent predictive or biological effects even in the presence of strong pathology signals. Complementary work has shown that multimodal feature integration improves LNM prediction performance in CRC [38,42], consistent with the hierarchical architecture observed here.

The addition of permutation importance and SHAP analysis strengthened the interpretation of the pathology-augmented models. LVI and age remained the two leading predictors across all three importance methods and both tree-based algorithms, indicating that these principal rankings were not dependent on impurity-based importance alone. In contrast, individual iron-handling variables showed less stable rankings and generally small permutation effects. Their non-zero Gini and SHAP contributions should therefore be interpreted as potentially correlated or interaction-dependent signals rather than evidence of strong independent predictive effects.

### 4.5. Biological Plausibility of Ferroptosis-Related Signals in Invasive and Metastatic Phenotypes

Our findings support a biologically plausible association between ferroptosis-related iron dysregulation, lymphovascular invasion, and nodal metastasis risk, rather than demonstrating a definitive causal cascade. LVI-positive patients showed a substantially higher N+ rate than LVI-negative patients (69.5% vs. 18.0%; relative risk = 3.86×), confirming LVI as a strong clinicopathological correlate of nodal metastasis. The pathway summary and biomarker distributions provided in Supplementary Figures S5 and S6 are therefore interpreted as exploratory evidence supporting a ferroptosis-related invasive phenotype, not as proof that ferroptosis directly causes LVI or lymphatic dissemination. This interpretation is consistent with experimental evidence that lymphatic environments may protect disseminating cancer cells from ferroptotic stress during metastatic spread [32], while our clinical data remain associative.

Previous preoperative LNM studies suggest that combining complementary predictor domains, such as imaging and clinical variables, may improve nodal-risk estimation [35,36,43]. However, in the present study, formal paired AUC comparisons did not demonstrate a statistically significant or consistent incremental benefit from adding iron-handling indices to the baseline LNM panel. The observed LVI performance changes were numerical and model-dependent and should therefore be considered exploratory. Although integrative machine-learning frameworks combining genomic, transcriptomic, and clinical features provide a methodological precedent for multimodal analysis [42], they do not establish the clinical utility of the present internally validated models.

The biological plausibility of a ferroptosis–CRC link extends beyond generic cancer biology: the colonic mucosa is chronically exposed to unabsorbed dietary heme iron, which catalyzes luminal lipid peroxidation and generates localized ferroptotic stress [44]. Epidemiological data consistently associate high red-meat and heme-iron intake with CRC risk—a relationship sufficiently robust for the IARC to classify processed meat as a Group 1 carcinogen, with heme-iron-mediated oxidative damage as a key proposed mechanism [45]. Furthermore, iron-deficiency anemia is one of the most frequent presenting features of colon malignancies, particularly right-sided tumors, indicating that systemic iron indices inherently reflect tumor–host metabolic interactions in this organ [46]. Accordingly, the evaluated laboratory panel may reflect a mixture of systemic iron availability, anemia, inflammation, nutritional status, blood loss, and tumor–host metabolic interactions. Although these processes overlap with ferroptosis-relevant biology, the present data cannot determine which biological mechanism underlies the observed model associations.

### 4.6. Limitations

This study has several limitations. First, the clinical models were developed in a retrospective single-center cohort and assessed through internal validation. Although cross-validation and bootstrap confidence intervals were used, external multicenter validation remains necessary before clinical implementation. Second, ferritin, serum iron, CEA, and CA19-9 had substantial missingness. Sensitivity analyses supported general model stability, but residual missing-not-at-random bias and imputation-related uncertainty cannot be excluded. Third, the moderate discrimination and limited calibration of the preoperative models require cautious interpretation, while the pathology-augmented model should be considered explanatory because it includes postoperative variables. Fourth, routine iron-handling indices are influenced by anemia, inflammation, nutrition, and other clinical factors and are not direct measures of tumor ferroptosis. Fifth, the transformer-assisted workflow ranked a predefined candidate space and should be regarded as literature-based prioritization rather than de novo gene discovery. Finally, TCGA-COAD and GSE39582 are bulk-tissue datasets and cannot resolve cell-specific or spatial ferroptosis signals. Prospective multicenter cohorts with standardized laboratory collection, external clinical validation, and single-cell or spatial transcriptomic approaches are therefore required.

## 5. Conclusions

This study suggests that routinely measured iron-handling indices may contain exploratory model-relevant information for LVI and LNM; however, their contribution was model-dependent and substantially limited by missing data. The integrated “AI → transcriptomics → clinical ML” framework identified a biologically coherent ferroptosis-related axis that is remodeled in tumors and associated with invasion-related phenotypes. While postoperative pathology variables, particularly LVI, provide strong explanatory information after resection, they are not available for preoperative deployment. In contrast, routine ferroptosis-linked iron-handling indices offer a low-cost and clinically accessible adjunct for preoperative risk stratification, pending external validation.

## Data Availability

The datasets generated and/or analyzed during the current study are available from the corresponding author upon reasonable request. Publicly available datasets were also utilized, including the TCGA-COAD cohort and the GSE39582 dataset from the Gene Expression Omnibus (GEO) database. (TCGA-COAD: https://portal.gdc.cancer.gov/ accessed on 24 June 2026, GSE39582: https://www.ncbi.nlm.nih.gov/geo/query/acc.cgi?acc=GSE39582 accessed on 24 June 2026).

## Supplementary Materials

The following supporting information can be downloaded at https://www.mdpi.com/article/10.3390/metabo16080557/s1.

## Abbreviations

The following abbreviations are used in this manuscript:

LNM	Lymph Node Metastasis
CRC	Colon Cancer
TCGA-COAD	The Cancer Genome Atlas Colon Adenocarcinoma
LVI	Lymphovascular Invasion
CT	Computed Tomography
MRI	Magnetic Resonance Imaging
GPX4	Glutathione Peroxidase 4
SLC7A11	Solute Carrier Family 7 Member 11
ACSL4	Acyl-CoA synthetase long-chain family member 4
EMT	Epithelial-mesenchymal transition
MCV	Mean corpuscular volume
TIBC	Total-iron binding capacity
AI	Artificial Intelligence
ML	Machine Learning
