# Supplementary material for: AI-Guided Ferroptosis Biomarker Discovery and Routine Laboratory–Based Machine Learning for Predicting Nodal Metastasis in Colon Cancer
Source: Metabolites. 2026 Aug 6;16(8):557. doi: 10.3390/metabo16080557 (PMC13515239; doi:10.3390/metabo16080557)
Supplement: Supplementary file 1 [file metabolites-16-00557-s001.zip › Supplementary_file_R.pdf]

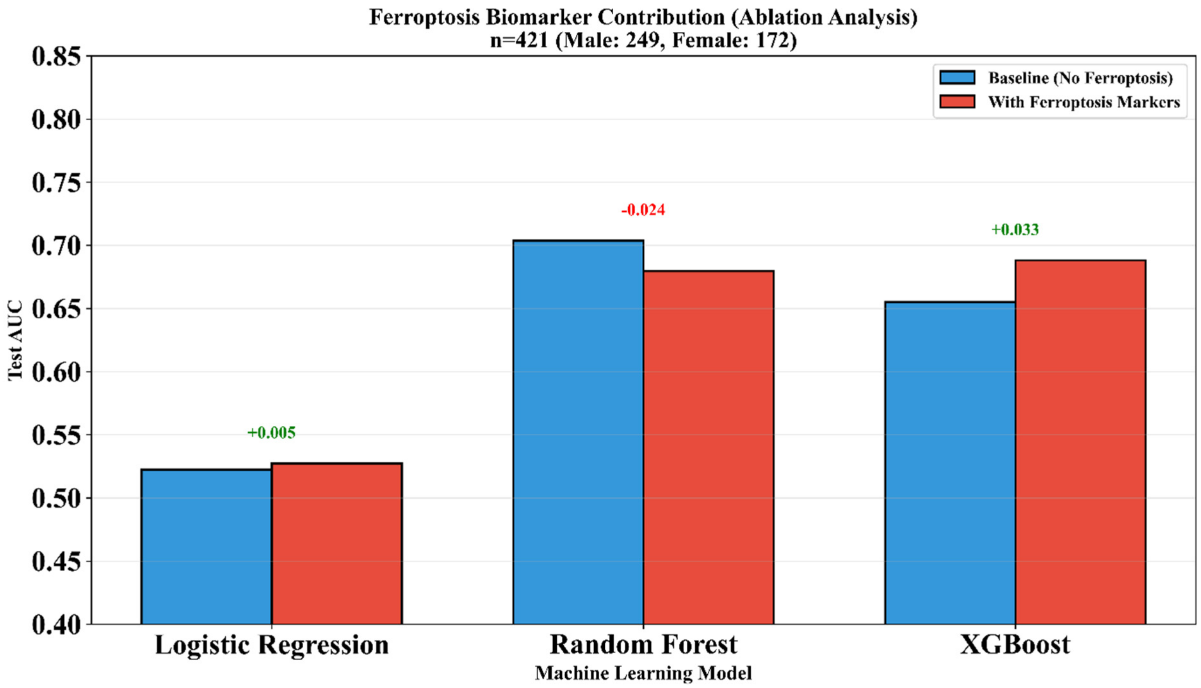

Supplementary Figure S1. Ferroptosis biomarker contribution (ablation analysis). Test AUC comparison for models trained without versus with ferroptosis-related markers, illustrating the incremental predictive contribution of the ferroptosis feature block across classifiers.

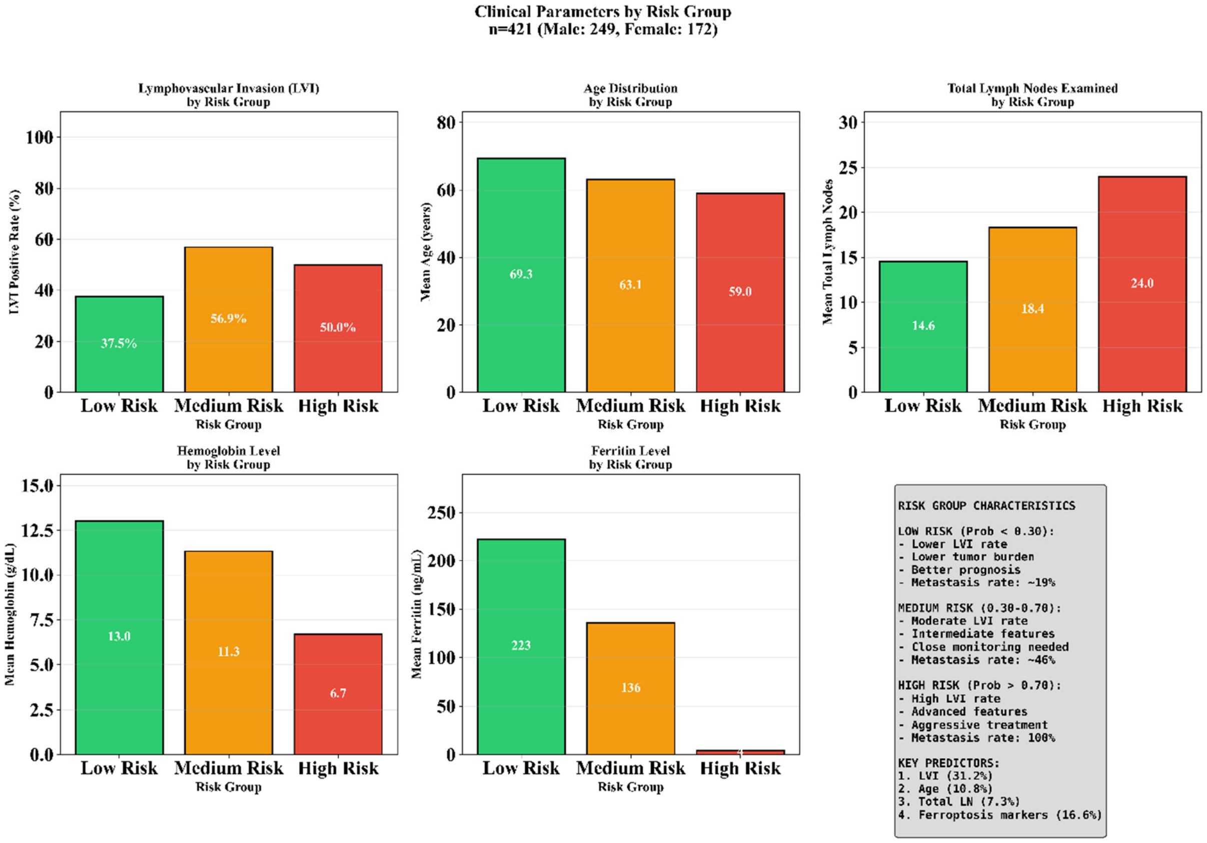

Supplementary Figure S2. Clinical parameter profiles across predicted risk groups. Group-wise summaries of key clinicopathological and laboratory variables across low-, intermediate-, and high-risk strata derived from the preoperative model.

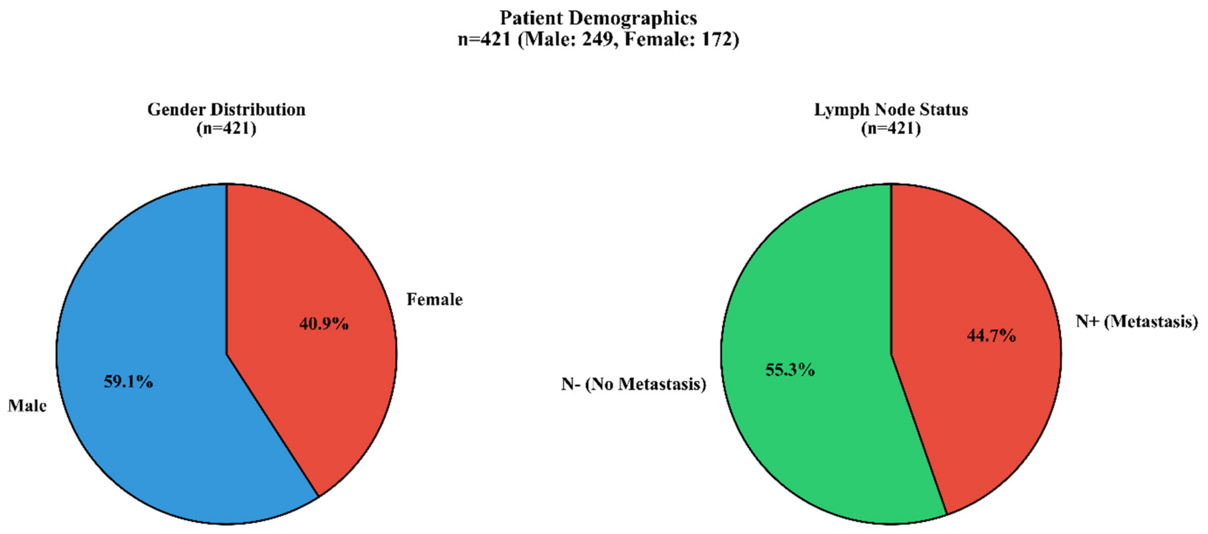

Supplementary Figure S3. Patient demographics and lymph node status (n = 421). Distribution of sex and nodal metastasis status in the clinical cohort used for machine-learning analyses.

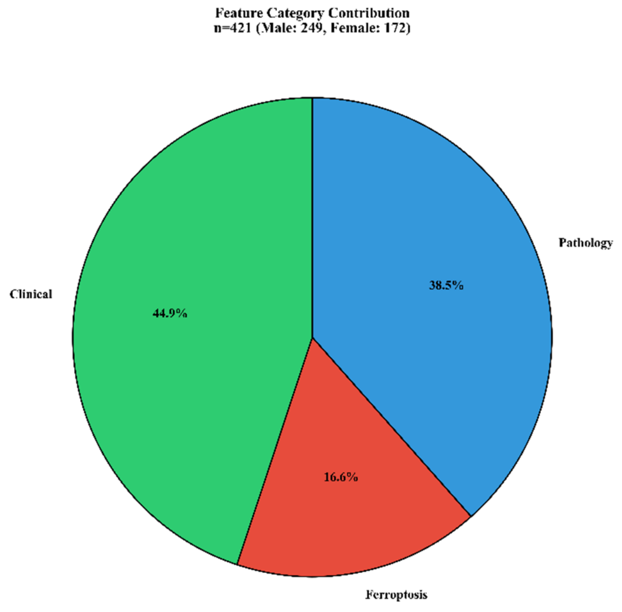

Supplementary Figure S4. Feature-category contribution. Proportional contribution of feature categories (clinical, pathology, ferroptosis) to the overall feature-importance signal, computed from the trained ensemble model.

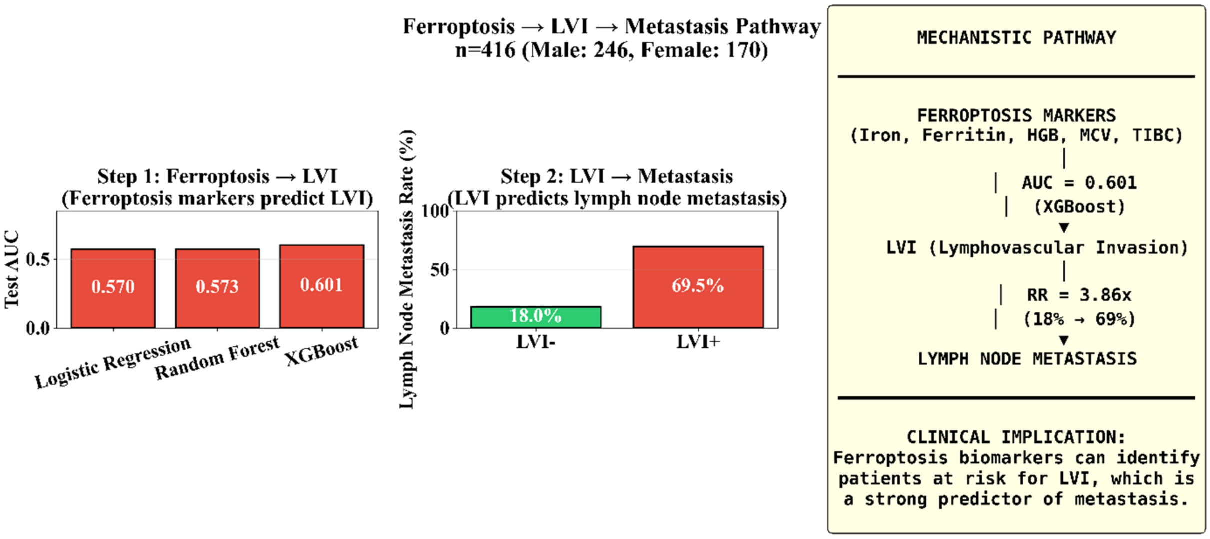

Supplementary Figure S5. Ferroptosis → LVI → metastasis pathway summary (n=416). A two-step schematic showing (i) prediction of LVI using ferroptosis markers and (ii) increased observed metastasis rate in LVI-positive patients, presented as a compact mechanistic/clinical interpretation panel.

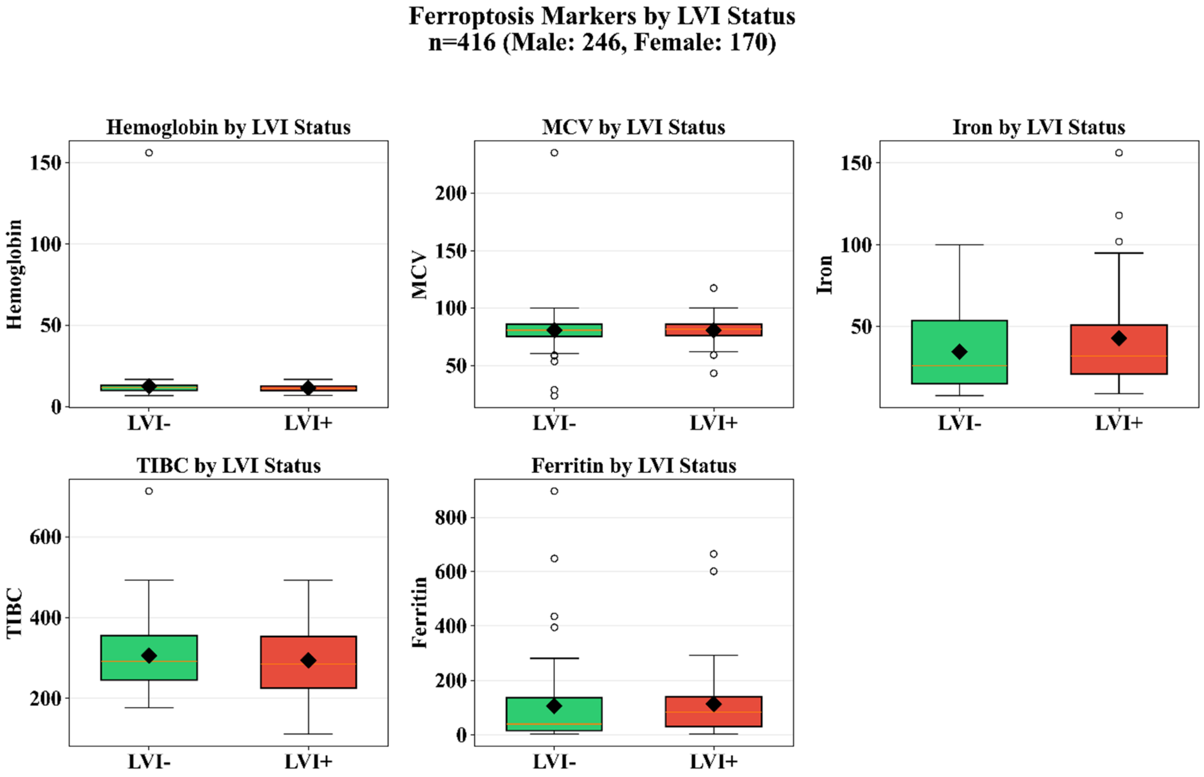

Supplementary Figure S6. Ferroptosis biomarkers by LVI status (n=416). Boxplot distributions of hemoglobin, MCV, iron, TIBC, and ferritin stratified by LVI status; boxes represent the interquartile range with median line, whiskers indicate dispersion, and points denote outliers.

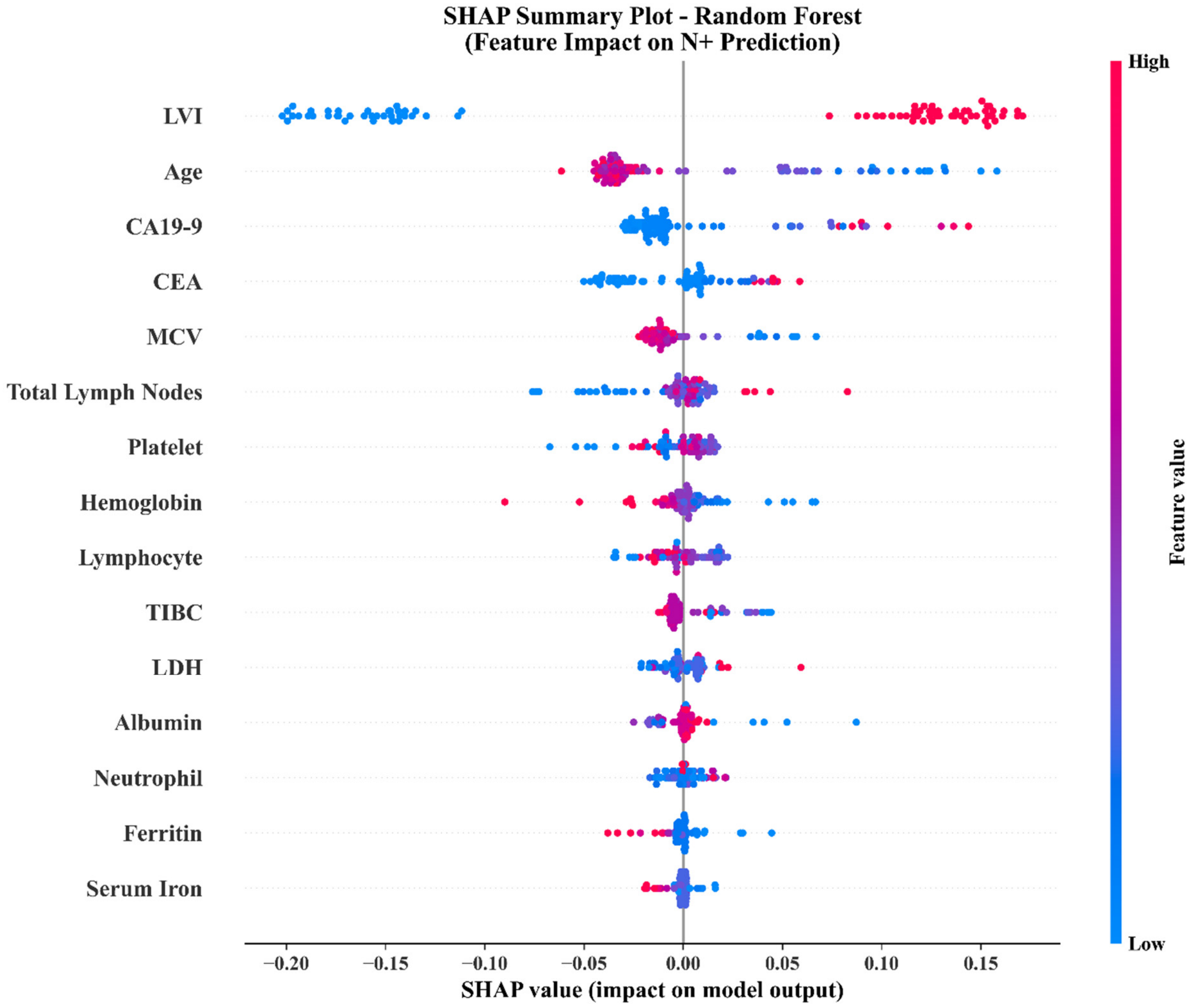

Supplementary Figure S7. SHAP-based interpretation of the pathology-augmented Random Forest model for lymph node metastasis prediction. The SHAP summary plot was generated using the held-out test set (n = 85) and the same 15-feature pathology-augmented model reported in Figure 7C. Features are ordered according to their mean absolute SHAP values. Each point represents one patient, point color indicates the corresponding feature value, and positive SHAP values shift the prediction toward lymph node-positive (N+) status. Lymphovascular invasion (LVI) was the dominant contributor, followed by age. LVI presence, younger age, and higher CA19-9 values generally shifted predictions toward N+ status. SHAP values describe model behavior and should not be interpreted as causal effects.

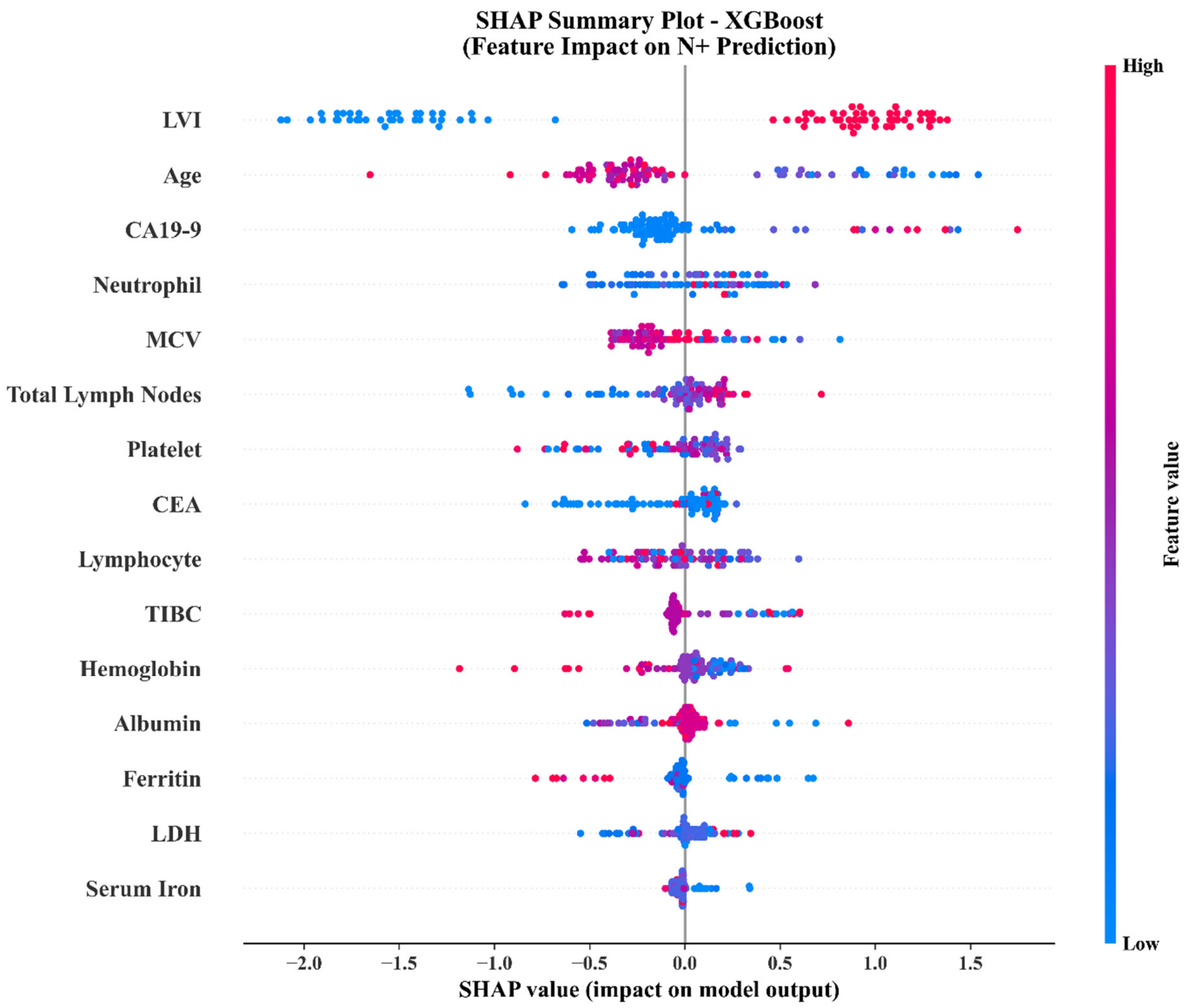

Supplementary Figure S8. SHAP-based interpretation of the pathology-augmented XGBoost model for lymph node metastasis prediction. The SHAP summary plot was generated using the held-out test set (n = 85) and the same 15-feature pathology-augmented model used in the primary analysis. Features are ordered according to their mean absolute SHAP values. Each point represents one patient, point color indicates the corresponding feature value, and positive SHAP values shift the prediction toward lymph node-positive (N+) status. Lymphovascular invasion (LVI) and age were the two leading contributors. LVI presence and younger age generally shifted predictions toward N+ status, while the remaining predictors showed smaller and more heterogeneous contributions. SHAP values represent model-specific associations and do not establish causal relationships.

Supplementary Methods S1. Composite score calculation for AI-guided biomarker prioritization

For literature-derived biomarker prioritization, each candidate gene was assigned a composite score calculated from five normalized components: evidence strength, statistical significance, clinical evidence density, effect magnitude, and cross-study reliability. All components were normalized to a 0–100 scale before weighting. Evidence strength received the highest weight

because it integrated both biomedical semantic relevance and ferroptosis-specific textual evidence. Statistical significance and clinical evidence density were weighted next to prioritize candidates supported by reproducible enrichment and patient-related evidence. Effect magnitude and reliability were included to reduce the influence of isolated or inconsistent signals. The resulting DL-Top 15 list was used only as a candidate-prioritization framework for downstream TCGA-COAD and GSE39582 validation.

Supplementary Table S1. Composite score components used for biomarker prioritization

Component	Weight	Purpose
Evidence strength	30%	Captures ferroptosis-specific textual and semantic relevance
Statistical significance	25%	Reflects FDR-corrected enrichment strength
Clinical evidence density	20%	Prioritizes candidates supported by clinical/patient-related evidence
Effect magnitude	15%	Captures standardized signal strength
Cross-study reliability	10%	Reflects consistency across retrieved studies

Supplementary Table S2. Missingness of preoperative predictors according to lymph-node metastasis status in the LNM cohort (n = 421).

Missingness rates were compared between patients without lymph-node metastasis (N−, n = 233) and those with lymph-node metastasis (N+, n = 188) using Fisher’s exact test. Odds ratios greater than 1 indicate higher odds of a missing measurement in the N+ group. None of the evaluated missingness indicators was significantly associated with LNM status.

Predictor	Overall missing, n (%)	N− missing, n (%)	N+ missing, n (%)	OR	p-value
Age	0 (0.0)	0 (0.0)	0 (0.0)	—	1.000
CEA	199 (47.3)	105 (45.1)	94 (50.0)	1.22	0.327
CA19-9	228 (54.2)	128 (54.9)	100 (53.2)	0.93	0.768
Neutrophil	27 (6.4)	11 (4.7)	16 (8.5)	1.88	0.160
Lymphocyte	27 (6.4)	15 (6.4)	12 (6.4)	0.99	1.000
Platelet	1 (0.2)	1 (0.4)	0 (0.0)	—	1.000
Albumin	164 (39.0)	85 (36.5)	79 (42.0)	1.26	0.269
LDH	169 (40.1)	90 (38.6)	79 (42.0)	1.15	0.486
Hemoglobin	117 (27.8)	60 (25.8)	57 (30.3)	1.25	0.325
MCV	1 (0.2)	1 (0.4)	0 (0.0)	—	1.000
Serum iron	304 (72.2)	167 (71.7)	137 (72.9)	1.06	0.827
TIBC	297 (70.5)	166 (71.2)	131 (69.7)	0.93	0.748
Ferritin	315 (74.8)	174 (74.7)	141 (75.0)	1.02	1.000

Abbreviations: CEA, carcinoembryonic antigen; CA19-9, carbohydrate antigen 19-9; LDH, lactate dehydrogenase; MCV, mean corpuscular volume; TIBC, total iron-binding capacity; OR, odds ratio; LNM, lymph-node metastasis.

Supplementary Table S3. Held-out test-set performance and bootstrap confidence intervals for LNM models. Values represent point estimates with percentile-based 95% confidence intervals obtained from 1,000 nonparametric bootstrap resamples of the held-out test set (n = 85; N+ = 38, N− = 47). The pathology-augmented models contain postoperative variables and are presented as explanatory benchmarks rather than preoperative prediction tools.

**Feature configuration**	**Model**	**AUC (95% CI)**	**Accuracy (95% CI)**	**Precision (95% CI)**	**Sensitivity (95% CI)**	**F1-score (95% CI)**
Preoperative baseline	Logistic regression	0.522 (0.390–0.653)	0.612 (0.518–0.718)	0.600 (0.409–0.800)	0.395 (0.237–0.568)	0.476 (0.310–0.623)
Preoperative baseline	Random Forest	0.704 (0.588–0.808)	0.635 (0.529–0.729)	0.600 (0.433–0.769)	0.553 (0.395–0.706)	0.575 (0.438–0.700)
Preoperative baseline	XGBoost	0.655 (0.533–0.771)	0.624 (0.518–0.729)	0.588 (0.424–0.750)	0.526 (0.367–0.683)	0.556 (0.400–0.676)
Baseline + iron-handling indices	Logistic regression	0.527 (0.398–0.651)	0.588 (0.482–0.694)	0.556 (0.367–0.743)	0.395 (0.242–0.553)	0.462 (0.300–0.606)
Baseline + iron-handling indices	Random Forest	0.680 (0.559–0.785)	0.600 (0.494–0.706)	0.567 (0.387–0.750)	0.447 (0.289–0.606)	0.500 (0.339–0.630)
Baseline + iron-handling indices	XGBoost	0.688 (0.560–0.791)	0.682 (0.588–0.776)	0.690 (0.517–0.846)	0.526 (0.362–0.686)	0.597 (0.444–0.714)
Pathology-augmented explanatory model	Logistic regression	0.775 (0.664–0.875)	0.765 (0.671–0.847)	0.705 (0.568–0.833)	0.816 (0.700–0.935)	0.756 (0.642–0.851)
Pathology-augmented explanatory model	Random Forest	0.818 (0.724–0.908)	0.800 (0.718–0.882)	0.756 (0.622–0.889)	0.816 (0.694–0.929)	0.785 (0.676–0.879)
Pathology-augmented explanatory model	XGBoost	0.791 (0.695–0.888)	0.718 (0.624–0.812)	0.684 (0.538–0.838)	0.684 (0.528–0.829)	0.684 (0.557–0.800)

Abbreviations: AUC, area under the receiver operating characteristic curve; CI, confidence interval; LNM, lymph node metastasis; N+, node-positive; N−, node-negative.

Supplementary Figure S9. Bootstrap confidence intervals for held-out test-set AUCs in LNM prediction. Diamonds represent test-set AUC point estimates, and horizontal lines represent percentile-based 95% confidence intervals obtained from 1,000 nonparametric bootstrap resamples of the held-out test set (n = 85; N+ = 38, N− = 47). The preoperative baseline model contained eight predictors, the iron-handling-augmented model contained 13 predictors, and the pathology-augmented explanatory model contained 15 predictors. The pathology-augmented configuration included postoperative LVI and total harvested lymph node count and was not intended for preoperative deployment.

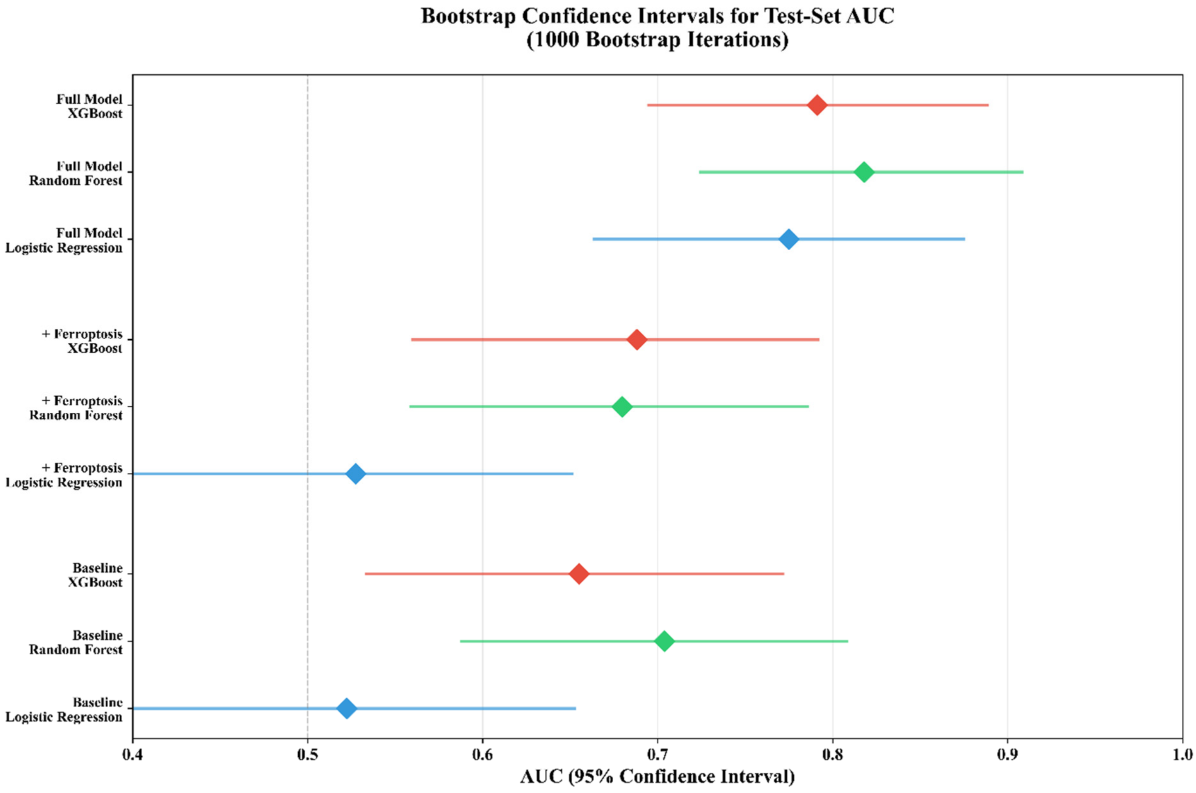

Supplementary Table S4. Formal evaluation of the incremental contribution of iron-handling indices to held-out LNM discrimination. Baseline and augmented AUCs were derived from paired predictions in the same held-out test set (n = 85; 38 N+ and 47 N−). Confidence intervals for ΔAUC were calculated using 10,000 paired bootstrap resamples. Two-sided paired DeLong tests were used for formal AUC comparisons. None of the comparisons was statistically significant.

Model	Baseline AUC	Augmented AUC	ΔAUC	Paired-bootstrap 95% CI	DeLong p
Logistic regression	0.522	0.527	+0.005	−0.052 to 0.058	0.853
Random Forest	0.704	0.680	−0.024	−0.077 to 0.026	0.341
XGBoost	0.655	0.688	+0.033	−0.034 to 0.104	0.348
